# The relationship between Social Determinants of Health (SDoH) and death from cardiovascular disease or opioid use in counties across the United States (2009–2018)

**DOI:** 10.1186/s12889-022-12653-8

**Published:** 2022-02-04

**Authors:** Pavani Rangachari, Anuraag Govindarajan, Renuka Mehta, Dean Seehusen, R. Karl Rethemeyer

**Affiliations:** 1 Department of Interdisciplinary Health Sciences, Augusta University, 987 St. Sebastian Way, Augusta, GA 30912 USA; 2 Department of Family Medicine, Augusta University, 987 St. Sebastian Way, Augusta, GA 30912 USA; 3 College of Computing, Georgia Institute of Technology, Atlanta, GA 30332 USA; 4 Department of Pediatrics, Augusta University, Augusta, GA 30912 USA; 5 Department of Family Medicine, Augusta University, Augusta, GA 30912 USA; 6 College of Social and Behavioral Sciences, University of Massachusetts, Amherst, MA 01003 USA

**Keywords:** Social Determinants of Health (SDoH), Socio-economic context, Cardiovascular mortality, Opioid use mortality, County-level determinants of health

## Abstract

**Background:**

Death from cardiovascular disease (CVD) has been a longstanding public health challenge in the US, whereas death from opioid use is a recent, growing public health crisis. While population-level approaches to reducing CVD risk are known to be effective in preventing CVD deaths, more targeted approaches in high-risk communities are known to work better for reducing risk of opioid overdose. For communities to plan effectively in addressing both public health challenges, they need information on significant community-level (vs individual-level) predictors of death from CVD or opioid use. This study addresses this need by examining the relationship between 1) county-level social determinants of health (SDoH) and CVD deaths and 2) county-level SDoH and opioid-use deaths in the US, over a ten-year period (2009–2018).

**Methods:**

A single national county-level ten-year ‘SDoH Database’ is analyzed, to address study objectives. Fixed-effects panel-data regression analysis, including county, year, and state-by-year fixed effects, is used to examine the relationship between 1) SDoH and CVD death-rate and 2) SDoH and opioid-use death-rate. Eighteen independent (SDoH) variables are included, spanning three contexts: socio-economic (e.g., race/ethnicity, income); healthcare (e.g., system-characteristics); and physical-infrastructure (e.g., housing).

**Results:**

After adjusting for county, year, and state-by-year fixed effects, the significant county-level positive SDoH predictors for CVD death rate were, median age and percentage of civilian population in armed forces. The only significant negative predictor was percentage of population reporting White race. On the other hand, the four significant negative predictors of opioid use death rate were median age, median household income, percent of population reporting Hispanic ethnicity and percentage of civilian population consisting of veterans. Notably, a dollar increase in median household income, was estimated to decrease sample mean opioid death rate by 0.0015% based on coefficient value, and by 20.05% based on effect size.

**Conclusions:**

The study provides several practice and policy implications for addressing SDoH barriers at the county level, including population-based approaches to reduce CVD mortality risk among people in military service, and policy-based interventions to increase household income (e.g., by raising county minimum wage), to reduce mortality risk from opioid overdoses.

**Supplementary Information:**

The online version contains supplementary material available at 10.1186/s12889-022-12653-8.

## Introduction

Cardiovascular disease (CVD) has remained a leading cause of death and disability in the United States (US) for over 80 years. In 2019, 840,768 people died from CVD, accounting for nearly 30% of all deaths in the US. CVD is estimated to cost the nation over $350 billion per year [1–3]. The opioid use crisis, on the other hand, is a more recent and escalating public health challenge in the US. In 2019 alone, drug-related overdoses (including prescription opioids and illicit drugs), claimed the lives of over 70,000 people in the US [4, 5]. The total economic burden of prescription opioid misuse alone is estimated to be nearly $80 billion per year, including direct healthcare costs, lost productivity, addiction treatment, and criminal justice involvement [6, 7].

While mortality from CVD and mortality from opioid use could be perceived as disparate societal issues, both are significant public health challenges that any given community (e.g., county of residence within the US) may be experiencing concurrently. Not surprisingly therefore, over the past ten-plus years, reducing the “community-level” risk of mortality from CVD and mortality from opioid overdose, have both been leading public health priorities of the US Department of Health and Human Services (DHHS) and the World Health Organization (WHO) [8–11]. For any given community concurrently experiencing both challenges, a logical first step towards addressing them, would be to understand the relationship between “community-level” social determinants of health and mortality from CVD or mortality from opioid use.

### Study purpose and conceptual framework

The WHO defines the social determinants of health (SDoH) as “the circumstances in which people are born, grow, live, work, and age, and the systems put in place to deal with illness” [12–14]. This definition reflects the view that health and illness are not distributed randomly throughout human society, nor are the resources and supports needed to prevent illness and its effects. Instead, they tend to cluster at the intersections of socio-economic, healthcare, and environmental forces. SDoH encompass the social and economic opportunities alongside the resources and supports available in people’s homes, neighborhoods, and communities. Evidence suggests that these factors influence health and illness by shaping barriers & facilitators to access to care and health-related behaviors [12]. Although experienced by individuals, SDoH exist at the community level. For example, the county of residence is widely regarded as an appropriate ‘unit of community’ for characterizing the SDoH context [12, 15]. Based on this rationale, some examples of SDoH context at the county-level include: the socio-economic context (e.g., race/ethnicity, education, income), the healthcare context (e.g., healthcare system characteristics), and the physical infrastructure context (e.g., housing) [12, 15]. This study seeks to examine the relationship between 1) county-level SDoH and CVD deaths and 2) county-level SDoH and opioid-use deaths, in the US, over a ten-year period (2009–2018).

### Current evidence on SDoH (related to CVD and opioid use mortality)

Investigating the relationship between community (county)-level SDoH and 1) mortality from CVD and 2) mortality from opioid use, would be significant from multiple perspectives of research, policy, and practice. From a research perspective, previous studies have demonstrated that CVD mortality at the community level, could be effectively reduced through “population-level approaches” to reduce risk through the primary healthcare system (e.g., increasing patient health knowledge and self-management skills to address modifiable risk factors such as diet, exercise, and alcohol consumption) [16–24]. On the other hand, studies have shown that mortality from opioid overdose at the community level, could be effectively reduced through “targeted approaches” to reduce risk among high-risk patient and provider groups, (e.g., improving patient access to treatment and recovery services and providing evidence-based guidelines for opioid provider prescriptions in the emergency and outpatient settings) [25–28].

At the same time, however, both streams of literature (on CVD and opioid use mortality) have acknowledged that the benefits of mortality reduction have not been shared equally across all socio-economic, racial, and ethnic groups in the US [29–36]. For example, Blacks have been found be 2 to 3 times more likely to die of heart disease compared with Whites. Lower levels of educational attainment and lower income have also been found to be associated with a higher prevalence of risk factors for CVD [30–33]. In regard to opioid use mortality, studies have found that compared to Hispanics, Whites are at elevated risk of death from opioid overdose and people living in poverty are more likely to die from this cause, compared to those who are not [34–36]. In other words, both streams of literature have emphasized that SDoH barriers have prevented the full benefits of mortality prevention and risk reduction initiatives from being realized in communities. Correspondingly, both literature streams have emphasized the importance of designing interventions to modify the SDoH context (to make individuals’ default behaviors healthy), in order to realize the full potential of prevention initiatives at the community level [29, 35]. This in turn implies that, the most significant future opportunities for reducing CVD and opioid use mortality at the community level, lie with addressing social determinants of health.

### Gaps in the SDoH literature (related to CVD and opioid use mortality)

Although considerable SDoH literature exists pertaining to both public health challenges, the preponderance of this literature, emanates from individual-level studies on SDoH, e.g., studies describing the risk of CVD death among Blacks and other racial/ethnic minorities relative to Whites; or the risk of death from opioid use among unemployed relative to employed individuals [34, 37]. By comparison, there is limited literature on the relationship between county-level SDoH and: 1) ‘death from CVD’ and 2) ‘death from opioid use.’ For example, is a one percentage point increase in county Hispanic population significant in predicting a lower (or higher) county CVD or opioid use death rate? This is different from asking if Hispanics have a lower (or higher) risk of CVD mortality relative to Whites.

Moreover, this type of data would be vital for understanding the general connection between the community SDoH context and serious adverse consequences of CVD or opioid use, regardless of time or place. It could also be impactful in bringing healthcare organizations and policymakers together (at the community level), to develop effective policies for modifying the SDoH context, while designing meaningful approaches to reduce the burden of mortality from CVD and opioid overuse. In addition to the dearth of community-level studies on the relationship between SDoH and mortality from CVD or opioid use, there are few studies that have examined the influence of SDoH on both ‘CVD deaths’ and ‘opioid use deaths.’ Existing studies that have paid concurrent attention to both public health challenges, have focused on the problem of cardiac arrests (deaths) resulting from opioid overuse, [38] which is distinctly different from the purpose and objectives of this study.

This study would have practical significance in addressing both gaps discussed above: 1) It would provide insight into the relationship between county-level SDoH and county-level deaths from a) CVD or b) opioid use; and 2) It would provide a foundation for understanding patterns of similarities/differences between significant county-level SDoH predictors of deaths from a) CVD and b) opioid use, which in turn, would enable communities to plan and prepare effectively to address both pressing public health challenges. In addition, the study would have conceptual significance in shedding light on the relationship between county-level SDoH and mortality, for both a longstanding public health challenge (CVD) and a recent, growing public health crisis (opioid use), in counties across the US. Since the effective “community-level” approach for mortality prevention may be different for CVD (population-wide approach) vs. opioid use (targeted approach), this study would provide insight into how community-level interventions could be tailored to modify the SDoH context based on the challenge at hand, i.e., ‘CVD deaths’ or ‘opioid use deaths’ or both, in counties (communities) across the US.

### Study objectives

In keeping with the broader purpose (described above), this study has four specific objectives:

1. Describe the distribution of ‘CVD deaths’ across regions, states, and counties of the United States over a ten-year period (2009–2018).
2. Describe the distribution of ‘opioid use deaths’ across regions, states, and counties of the United States over a ten-year period (2009–2018).
3. Examine the relationship between county-level social determinants of health (SDoH) and ‘CVD deaths’ in the United States from 2009–2018.
4. Examine the relationship between county-level social determinants of health (SDoH) and ‘opioid use deaths’ in the United States from 2009–2018.

As described in greater detail under Methodology, ‘CVD deaths’ (mortality) in this study, is defined to include death from heart disease and stroke, while ‘opioid use deaths’ (mortality) is defined to include death involving any opioid obtained legally (e.g., by prescription) or otherwise. By addressing the aforementioned four objectives, this study seeks to develop an understanding of significant county-level (vs. individual level) SDoH predictors of ‘CVD deaths’ and ‘opioid use deaths’ in the US, while also gaining insight into patterns of similarities and differences between county-level SDoH predictors of 1) death from CVD and 2) death from opioid use.

## Methodology

### Data sources

This study uses a single comprehensive US national database, “The Social Determinants of Health (SDoH) Database,” to address all four objectives. The SDoH database was created by the US Agency for Healthcare Research and Quality (AHRQ), with funding from the Patient-Centered Outcomes Research Trust Fund, and released for public use in the fall of 2020 [39]. The multi-year database curated from multiple publicly available federal data sources, is designed to include only “area-level” data elements within the US (e.g., at the county level or the zip-code area level). In other words, the database does not include any “individual-level” data elements. Examples of federal data sources from which the AHRQ SDoH data are derived include: the American Community Survey (ACS), the amfAR Opioid & Health Indicators Database (amfAR), the CDC Interactive Atlas for Heart Disease and Stroke (CDC Atlas), the CDC Wide-ranging ONline Data for Epidemiologic Research (CDC WONDER), and the US Census Bureau, among others. The database documentation includes the full listing of data sources [15].

The current version of the AHRQ SDoH county-level database includes data for all 3,226 US counties over a ten-year-period (2009–2018) for some SDoH variables, with less data available for other SDoH variables. Each annual county data file contains one record per county, uniquely identifiable through a five-digit state-county Federal Information Processing Standards (FIPS) code. The state in which the county is based is also included, as are basic data on county physical characteristics (land area, rural–urban continuum code, population density) and total weighted population.

### Limitations of the US AHRQ SDoH framework and database

In broad terms, the SDoH variables in the AHRQ database correspond to three contextual domains: 1) Socio-economic context (including age, gender, race/ethnicity, living conditions, social vulnerability index, segregation, workforce/employment, poverty, income, education, population demographic distribution); 2) Healthcare context (including healthcare system characteristics, health insurance status, healthcare access, quality, health behaviors, healthcare utilization, health status); and 3) Physical infrastructure context ( including environment, crime, housing, food access, and transportation). The AHRQ SDoH framework and variable-set are rather well-aligned with the widely-referenced World Health Organization (WHO) SDoH framework [12]. The WHO framework demonstrates how social, economic, and political factors such as income, education, occupation, gender, race, and ethnicity influence a person’s socio-economic position which in turn, plays a significant role in determining health outcomes. Correspondingly, the WHO framework considers the role of income, education, unemployment, food insecurity, and access to affordable health services (among other factors) in influencing health outcomes and health equity. As outlined above, these factors are also well represented in the AHRQ SDoH dataset. However, it must be noted that WHO provides a broader SDoH framework encompassing a wider array of forces and conditions shaping the conditions of daily living, including structural determinants of health inequities (encompassing macroeconomic policies, social polies, public policies, and cultural and societal values). The AHRQ SDoH framework is limited in its representation of this component of the WHO framework, which in turn could be viewed as a limitation. Over and above framework limitations, the AHRQ SDoH database has limitations of data availability, which directly impacted variable inclusion in this study (as elaborated in the next subsection).

### Criteria for exclusion of independent (SDoH) variables from the study

A majority of variables in the SDoH database were not available over all ten years [39]. As explained under ‘Empirical Approach’ (below), this study conducts fixed-effects regression analysis on ten-year county panel data to examine the relationship between county-level SDoH and death rates. This in turn, necessitated the exclusion of numerous SDoH variables that were not available across all ten years. It would be relevant to note that in addition to lack of availability of several SDoH variables across all ten years, a number variables in the dataset had varying availability varied over the ten-year period. For example, while data on health insurance status variables (e.g., percent of population on any private insurance) were missing for 2009–2012, data on external environment variables (e.g., Heat Index, air quality,) were missing for 2017–2018. Likewise, data on health status variables (e.g., percentage of adults reporting heavy drinking) were missing for 2009–2013 and 2018, while data on healthcare access (e.g., number of substance abuse treatment facilities offering medication-assistance treatment) were missing for 2009–2015, and data on healthcare use (e.g., number of hospital admissions) were missing for 2009 and 2018. Such issues in turn, precluded the option of using of shorter panel-data timeframes (e.g., 5 years vs. 10 years), to increase the prospects for data availability.

As indicated earlier, lack of data availability over the ten-year period, served as the first criterion for variable exclusion from this study. Additional file 1 provides a full listing of AHRQ SDoH variables alongside information on data availability. The second criterion for variable exclusion, was the statistical consideration of multi-collinearity among SDoH (independent) variables. The condition of multi-collinearity was assessed using correlation matrices (and resulting correlation coefficients and variance inflation factors), and was used to determine eligibility of SDoH variables for the fixed-effects regression analysis. The results of multi-collinearity assessments in turn, necessitated exclusion of seral SDoH variables from the analysis for addressing Objectives 3 and 4, due to either perfect collinearity or high collinearity with the panel (county) variable used in the fixed-effects regression analysis. This included several variables embodying physical characteristics of the county (i.e., land area, population density, and rural–urban continuum code) and other variables within the SDoH ‘physical-infrastructure’ domain (e.g., beer, wine, and liquor stores per 1000 people, full-service restaurants per 1000 people, and specialty food stores per 1,000 people). Additional file 1 includes information on the second exclusion criterion of multi-collinearity for all SDoH variables in the AHRQ dataset.

SDoH variables that were not excluded following application of the aforementioned two exclusion criteria, were included in the study. This included eighteen SDoH variables representing all three domains (‘socio-economic,’ ‘healthcare,’ and ‘physical infrastructure’), as outlined in the conceptual framework. It would be relevant to note that a majority of the included variables within the ‘socio-economic’ domain (e.g., gender, age, race, ethnicity, income, education, and unemployment), have been extensively researched in individual-level SDoH studies in the context of CVD and opioid use mortality, thereby enabling comparison between existing individual-level results and county-level SDoH results emanating from this study. Also, in keeping with the study purpose, the same set of SDoH variables was used to address both Objective 3 and Objective 4, to facilitate assessment of similarities and differences between significant county-level SDoH predictors of CVD and opioid use death rates. In summary, as indicated in Additional file 1 and Table 1 (below), following application of the two exclusion criteria, a total of 18 SDoH variables spanning three contextual domains, were included in the study.

**Table 1:** Summary Statistics for Dependent and Independent (SDoH) Variables of Interest (2009–2018)

Variable name	Variable label	Counties covered	Mean	S.D	Min	Max	N
**Dependent variables (outcome measures)**							
CDC_HEART_DISEASE_DEATH	Total cardiovascular disease (CVD) death rate per 100,000 population per year	3,152	250.7306	59.64733	56.7	645.6	31,017
CDC_OPIOID_DEATH	Total drug overdose death rate involving any opioid per 100,000 population per year	554	17.82371	15.24973	0.03	157.57	3,273
**Independent (SDoH) variables: Socio-economic context**							
ACS_PCT_FEMALE	Percentage of population that is female	3,226	50.03082	2.420218	19.16	62.64	32,208
ACS_MEDIAN_AGE	Median Age	3,226	40.46141	5.203414	18	67	32,208
ACS_PCT_BLACK	Percentage of population reporting Black race	3,223	8.940934	14.40663	0	87.412	32,205
ACS_PCT_WHITE	Percentage of population reporting White race	3,223	83.3241	16.78994	3.11	100	32,205
ACS_PCT_HISPAN	Percentage of population reporting Hispanic ethnicity	3,223	83.3241	19.15072	0	100	32,205
ACS_PCT_ASIAN	Percentage of population reporting Asian race	3,223	1.205131	2.624424	0	52.228	32,205
ACS_PCT_BACHELOR_DGR	Percentage of population with a bachelor's degree (ages 25 and over)	3,223	13.1801	5.419684	1.679	47.985	32,205
ACS_MEDIAN_HH_INCOME	Median household income	3,223	46,030.93	13,128.92	10,499	136,268	32,203
ACS_PCT_PERSON_INC99	Percentage of population with income to poverty ratio: < 1.00	3,223	16.9234	8.297911	0	65.715	32,204
ACS_PCT_UNEMPLOY	Percentage of population that was unemployed (ages 16 years and over)	3,223	4.737517	2.302576	0	30.132	32,204
ACS_PCT_GRP_QRT	Percentage of persons in institutionalized (correctional) group quarters	3,223	3.480389	4.601596	0	80.178	32,205
ACS_PCT_VA	Percentage of the civilian population consisting of veterans (ages 18 and over)	3,223	10.18986	3.068378	0	32.399	32,205
ACS_PCT_ARMED_FORCES	Percentage of civilian population in armed forces (ages 16 years and over)	3,223	0.322018	1.661381	0	81.247	32,204
**Independent (SDoH) variables: Healthcare context**							
AHRF_FED_HLTH_CNT	Number of Federally Qualified Health Centers	3,226	1.972197	5.914462	0	241	32,227
AHRF_RURAL_H_CLINIC	Number of rural health clinics	3,226	1.255283	2.035866	0	44	32,227
**Independent (SDoH) variables: Physical infrastructure context**							
ACS_TOTAL_HOUSEHOLD	Total number of households	3,223	36,457.85	110,690.9	22	3,306,109	32,205
ACS_PCT_RENTED_HH	Percentage of occupied housing units: rented	3,223	27.87347	8.065046	5.275	100	32,205
ACS_PCT_MOBILE_HOME	Percentage of housing units that are mobile homes	3,223	12.78241	9.492498	0	63.485	32,205

As discussed above, the limitations of the AHRQ SDoH framework and dataset are noteworthy. However, at this juncture, it would also be relevant to recognize that this study utilizes fixed-effects panel data regression techniques to address study objectives. This empirical approach is generally designed to include county- and year- fixed effects at a minimum (i.e., comprehensive controls for unobserved location and time-specific determinants of the dependent variable), which in turn, can help to gain insight into the general connection between dependent and independent variables, regardless of place or time. In other words, findings from fixed-effects analysis of multi-year county panel data, have potential to provide meaningful insights into the relationship between SDoH variables examined, and death from CVD or opioid use, regardless of the number of independent SDoH variables. Notably, other health economic research studies that have utilized similar methods and datasets (e.g., to examine the relationship between the macro-economic context and health behaviors using multi-year US county panel data) [40–46], have used as little as one independent variable (i.e., unemployment rate), as a proxy for macro-economic context. By comparison, this study is designed to examine the relationship between multiple SDoH variables, and death from CVD or opioid use in US counties over a ten-year period.

### Dependent and independent (SDoH) variables included in the study

Table 1 (below) provides summary statistics for all dependent and independent variables included in this study. Our two county-level dependent variables of interest were: 1) Total cardiovascular disease (CVD) death rate per 100,000 population per year (derived from CDC Atlas) [47]; and 2) Total drug overdose death rate involving any opioid per 100,000 population per year (derived from CDC WONDER) [48]. Mortality data in the CDC Atlas and CDC WONDER databases are based on death certificates for U.S. residents that are coded by the states and provided to the CDC’s National Center for Health Statistics (NCHS), through the Vital Statistics Cooperative Program. Each death certificate identifies underlying and multiple causes of death and includes demographic information [49, 50].

CVD deaths were identified by International Classification of Diseases, Tenth Revision (ICD-10) Multiple Cause of Death (MCOD) Codes: I00-I78 (Total Cardiovascular Disease); I00-I09, I11, I13, I20-I51 (All Heart Disease); I20-I25 (Coronary Heart Disease); I21-I22 (Acute Myocardial Infarction); I47-I49 (Cardiac Dysrhythmia); I50 (Heart Failure); I10-I15 (Hypertension); I60-I69 (All Stroke); I63, I65-I66 (Ischemic Stroke); and I60-I62 (Hemorrhagic Stroke). Likewise, opioid use deaths involving any opioid were identified by ICD-10 (MCOD) codes T40.0 (Opium); T40.1 (Heroin); T40.2 (Other opioids); T40.3 (Methadone); T40.4 (Other synthetic narcotics); and T40.6 (Other and unspecified narcotics).

### Descriptive statistics for objectives 1 & 2

We used a variety of descriptive methods to describe the distribution of CVD and opioid use deaths across counties, states, and regions within the US (Objectives 1 and 2). To begin with, every US county and state was assigned to a region. While prior studies have examined mortality for CVD and opioid use by US region, the vast majority have used the five-region classification (Northeast, Southwest, West, Southeast, and Midwest). In this study, we use the “ten federal regions” classification (below), to gain a more comprehensive understanding of the regional distribution of both mortality rates [51].

1. Region I: Connecticut, Maine, Massachusetts, New Hampshire, Rhode Island, Vermont
2. Region II: New Jersey, New York, Puerto Rico, US Virgin Islands
3. Region III: Delaware, District of Columbia, Maryland, Pennsylvania, Virginia, West Virginia
4. Region IV: Alabama, Florida, Georgia, Kentucky, Mississippi, North Carolina, South Carolina, Tennessee
5. Region V: Illinois, Indiana, Michigan, Minnesota, Ohio, Wisconsin
6. Region VI: Arkansas, Louisiana, New Mexico, Oklahoma, Texas
7. Region VII: Iowa, Kansas, Missouri, Nebraska
8. Region VIII: Colorado, Montana, North Dakota, South Dakota, Utah, Wyoming
9. Region IX: Arizona, California, Hawaii, Nevada, American Samoa, Guam, Northern Mariana Islands
10. Region X: Alaska, Idaho, Oregon, Washington

**Fig. 1:**
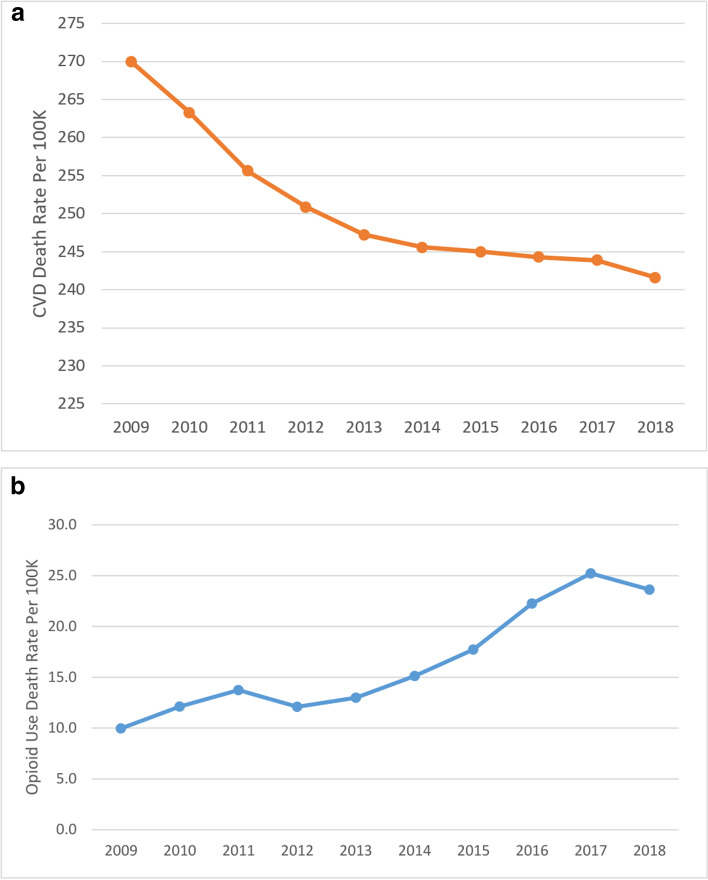
(Parts A & B): Trend in CVD and Opioid Use Death Rates in the US (2009–2018)

**Fig. 2:**
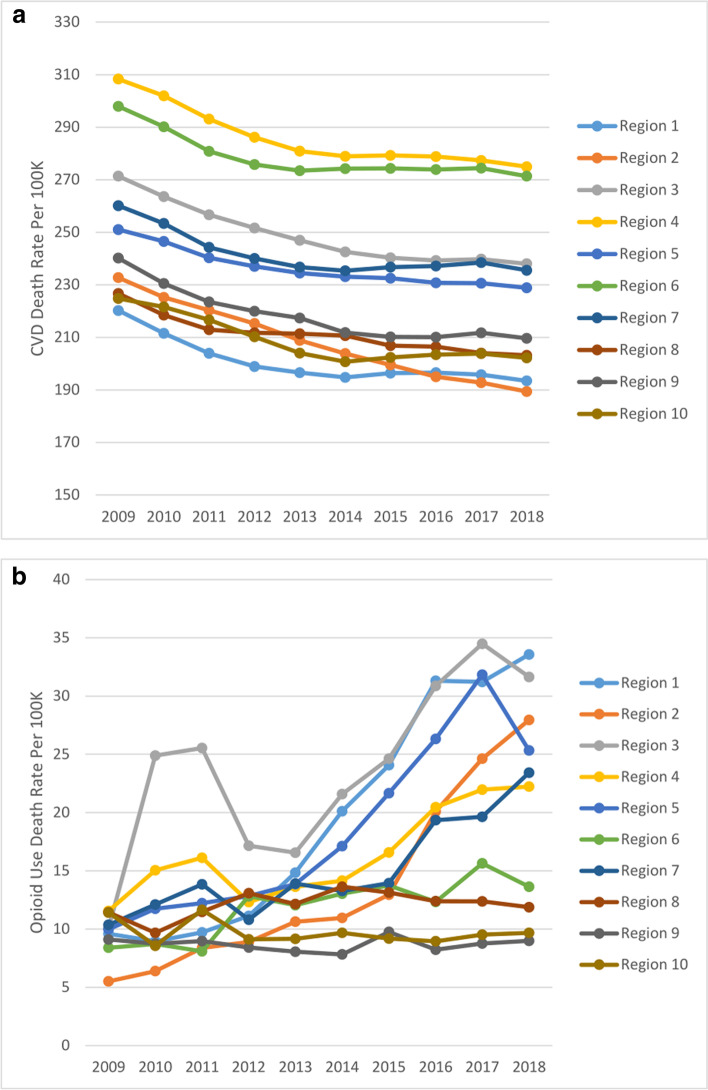
(Parts A & B): CVD and Opioid Use Death Rates by Region in the US (2009–2018)

**Fig. 3:**
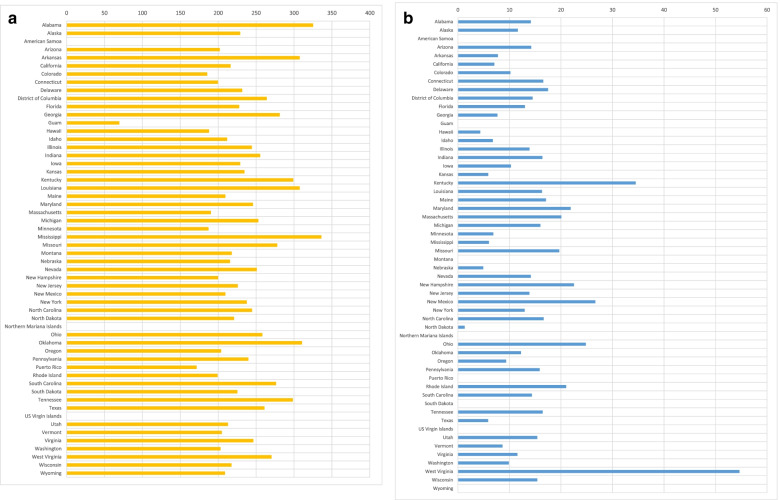
(Parts A & B): CVD and Opioid Use Death Rates by State in the US (2009–2018)

**Fig. 4:**
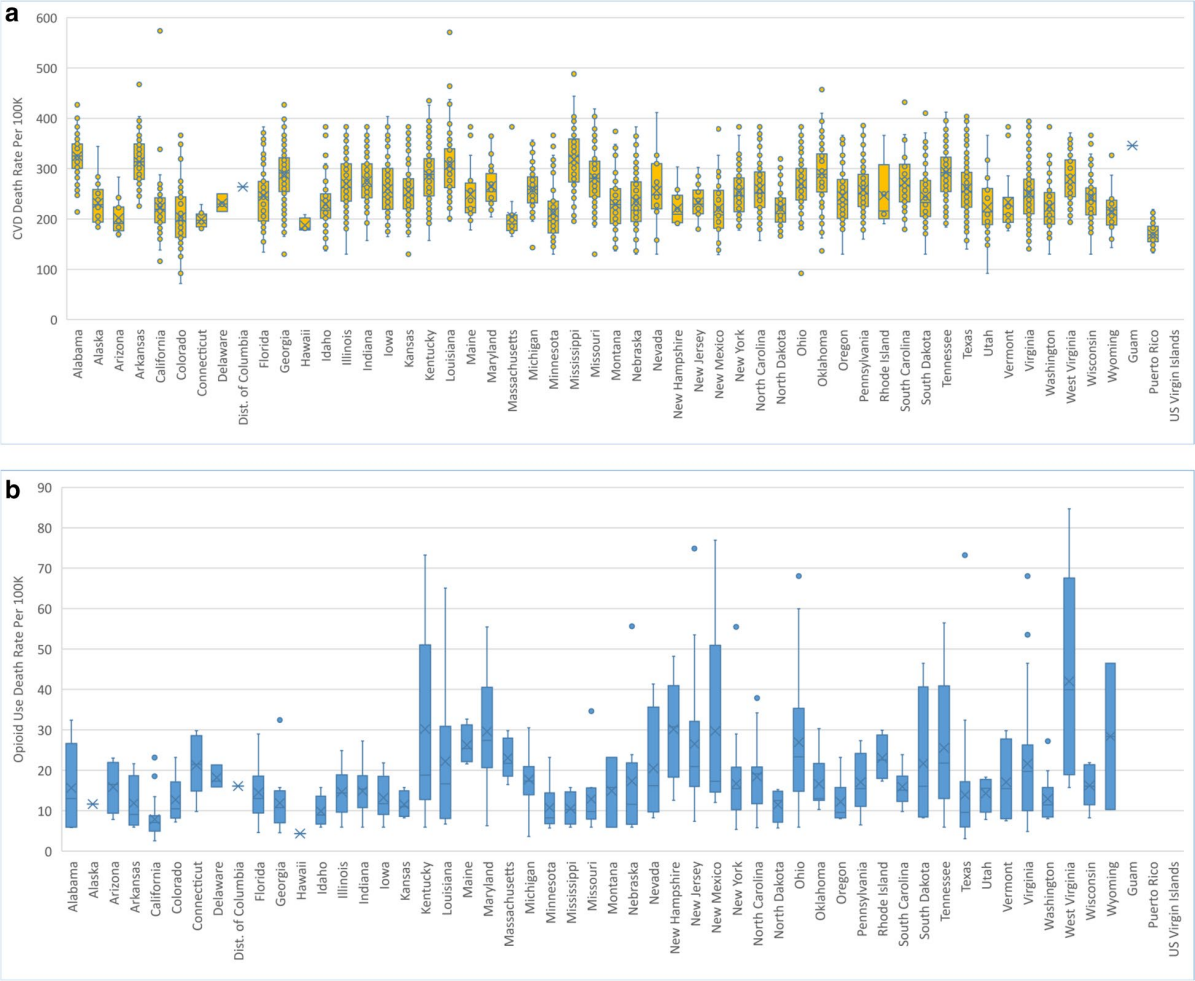
(Parts A & B): County-Level Variation in CVD and Opioid Use Death Rates by State (2009–2018)

To address Objectives 1 and 2, we examined the ten-year trend (2009–2018) in mean ‘CVD death rate’ and mean ‘opioid use death rate’ at the US national level (Fig. 1). We also examined the ten-year trend in the mean ‘CVD death rate’ and mean ‘opioid use death rate’ by region (Fig. 2) and the distribution of the overall mean ‘CVD death rate’ and the mean ‘opioid use death rate’ by state, for the full ten-year period (Fig. 3). Finally, we examined county-level disparities in the mean CVD and opioid use death rate within each state (over the full ten-year period) (Fig. 4).

### Empirical approach for objectives 3 and 4

We performed a county-level regression analysis of the relationship between SDoH and 1) ‘CVD death rate per 100,000’ and 2) ‘opioid use death rate per 100,000’ over a ten-year period (2009–2018), in the US. All statistical analysis was performed on STATA® Version 14. Our main regression specification took the form shown in Eq. 1 below:

1 $${\mathrm{Y}}_{\mathrm{jt}}={\upbeta }_{1}{\mathrm{X}}_{1,\mathrm{ jt}}+\dots +{\upbeta }_{\mathrm{k}}{\mathrm{X}}_{\mathrm{k},\mathrm{ jt}}+{\mathrm{\varphi }}_{\mathrm{j}}+{\updelta }_{\mathrm{t}}+{\upmu }_{\mathrm{st}}+{\upepsilon }_{\mathrm{jt}}$$

where the dependent variable ${\mathrm{Y}}_{\mathrm{jt}}$ is the death rate from ‘CVD’ or ‘opioid use’ in county $\mathrm{j}$ and year $\mathrm{t}$; ${\mathrm{X}}_{1\dots \mathrm{k},\mathrm{ jt}}$ represents the SDoH independent variables and ${\upbeta }_{1\dots \mathrm{k}}$ represents the coefficient for independent variables. We include county and year fixed-effects (${\mathrm{\varphi }}_{\mathrm{j}}$ and ${\updelta }_{\mathrm{t}}$) in all models to control for potential confounding factors that may vary across counties but are fixed over time (e.g., local policies or long-term effects of natural disasters like floods or hurricanes), as well as determinants of mortality that may vary nationally over time (e.g., national economic policies). One concern may be that local policies influencing mortality from CVD or opioid use may have changed in ways that are spuriously correlated with the SDoH variables. Of particular relevance would be Medicaid policies, prescription drug monitoring programs or medical marijuana legalization policies, all of which occur at the state rather than the county level. Therefore, our preferred specifications include state-by-year fixed effects (${\upmu }_{\mathrm{st}}$), in addition to county and year fixed-effects. We report results separately for 1) county and year fixed effects, 2) county, year, and state-by-year fixed effects. In doing so, our models are designed to include comprehensive controls for location and time-specific determinants of mortality.

As part of robustness checks, we control for heteroscedasticity (unequal error variance) by specifying robust standard errors in all our models. Our tables display robust standard errors (next to the coefficients) clustered at the county level, which is the level of variation for our SDoH independent (predictor) variables. All our regression models also control for weighted county population, enabling us to obtain nationally representative treatment effects. We used the Hausman Test to confirm the appropriateness of fixed effects vs random effects regression modeling for our panel data. All our regression models were significant based on F-statistic (*p* < 0.05), indicating that our models provided a better fit to the data than a model containing no independent variables. The STATA outputs for all reported model specifications are included in the supplementary materials for reference.

## Results

### Descriptive results for objectives 1 & 2

As indicated in Table 1, the mean county CVD death rate in our sample was 250.73 per 100,000 population per year, while the mean county opioid use death rate was 17.82 per 100,000 population per year. Fig. 1 shows the US (national) ten-year trend (2009–2018) in 1) mean county ‘CVD death rate’ (Part A) and 2) mean county ‘opioid use death rate’ (Part B). As indicated in the figure, the mean county CVD death rate in the US declined steadily from 269.98 per 100,000 in 2009 to 241.59 per 100,000 in 2018, a 11% decline. By contrast, the mean county opioid use death rate in the US rose sharply from 9.96 per 100,000 in 2009 to 23.63 per 100,000 in 2018, a 137% increase.

Figure 2 shows the ten-year trend in the mean county ‘CVD death rate’ (Part A) and mean county ‘opioid use death rate’ (Part B), by region. Both parts of the figure also include bar graphs depicting the overall ten-year average mean county death rates by region for CVD (Part A) and opioid use (Part B). The trend chart for CVD shows that Region 2 (New Jersey, New York, Puerto Rico, US Virgin Islands) experienced the biggest decline of 18.6% in CVD death rate from 232.71 per 100,000 in 2009 to 189.39 per 100,000 in 2018, while Region 5 (Illinois, Indiana, Michigan, Minnesota, Ohio, Wisconsin) had the smallest decline in CVD death rate of 8.8% from 251.02 per 100,000 in 2009 to 228.83 per 100,000 in 2018. On the other hand, Region 2 (New Jersey, New York, Puerto Rico, US Virgin Islands) experienced the biggest increase in opioid use death rate of 407.5% from 5.51 per 100,000 in 2009 to 27.95 per 100,000 in 2018, while both Region 9 (Arizona, California, Hawaii, Nevada, American Samoa, Guam, Northern Mariana Islands) and Region 10 (Alaska, Idaho, Oregon, Washington) experienced a decline in opioid use death rates of 1.3% and 15.2% respectively, with opioid use death rates for Region 10 declining from 11.41 per 100,000 in 2009 to 9.68 per 100,000 in 2018.

The bar graphs in Fig. 2 Part A indicate that Region 4 (Alabama, Florida, Georgia, Kentucky, Mississippi, North Carolina, South Carolina, Tennessee) had the highest average mean county CVD death rate of 285.94 per 100,000 over the ten-year period, while Region 1 (Connecticut, Maine, Massachusetts, New Hampshire, Rhode Island, Vermont) had the lowest at 200.82 per 100,000. On the other hand, the bar graphs in Part B indicate that Region 3 (Delaware, District of Columbia, Maryland, Pennsylvania, Virginia, West Virginia) had highest average mean county opioid use death rate of 23.74 per 100,000; Region 1 had the second-highest opioid use death rate at 19.45 per 100,000, while Region 9 had the lowest at 8.69 per 100,000.

Figure 3 provides bar graphs depicting the overall ten-year average of mean county death rates by state or US territory (instead of region), for both CVD (Part A) and for opioid use (Part B). Part A shows that Mississippi had the highest average of mean county CVD death rate over the ten year period, at 335.85 per 100,000. Alabama followed at a close second of 324.86 per 100,000, followed by Oklahoma, Louisiana, and Arkansas at 310.31, 307.25, and 307.21 per 100,000, respectively. By contrast, Puerto Rico, Colorado Minnesota, Hawaii, and Massachusetts recorded the lowest average CVD death rates over the ten-year period at 171.21, 185.23, 186.99, 187.49, and 190.13 per 100,000, respectively. Fig. 3 Part B reveals that West Virginia had the highest average of mean county opioid use death rate over the ten year period, at 54.64 per 100,000, followed by Kentucky, New Mexico, Ohio, and New Hampshire at 34.51, 26.67, 24.83, and 22.53 per 100,000 respectively. By contrast, among states or territories that reported every year over the ten-year period, the lowest opioid death rates were recorded by Hawaii, Nebraska, Texas, Kansas, and Mississippi, at 4.34, 4.91, 5.87, 5.89, and 6.02 per 100,000 respectively.

Figure 4 depicts county-level disparities in the mean CVD and opioid use death rates within each state (over the entire ten-year period). California, Louisiana, and Mississippi had among the highest county-level disparities in CVD death rates, with California ranging from a low of 115.87 per 100,000 to a high of 573.75 per 100,000. Louisiana, from a low of 201.13 to 570.67 per 100,000, and Mississippi from a low of 195.48 to a high of 488.19. On the other hand, Kentucky, New Mexico, and West Virginia, had among highest county-level disparities for opioid use death rates, with Kentucky ranging from a low of 5.92 per 100,000 to a high of to 73.24 per 100,000, New Mexico ranging from a low of 12.06 per 100,000 to a high of 76.93 per 100,000, and West Virginia ranging from a low of 15.72 per 100,000 to a high of 84.78 per 100,000.

### Empirical results for objectives 3 & 4

Table 2 shows two county-level specifications for our first dependent variable of interest, i.e., CVD death rate. The first and second columns shows the first specification with only county and year fixed effects, while the third and fourth columns show the second specification which includes state-by-year fixed effects and corresponds to Eq. 1. For both specifications, the table shows the coefficients representing estimated effects and the corresponding robust standard errors, clustered at the county level. Consistent with other similar published studies, we report statistical significance for three alpha levels, *p* < 0.10, *p* < 0.05, and *p* < 0.01 [40–46]. As mentioned earlier, our preference is for the models that include state-by-year fixed effects. The coefficient estimates under specification 1 (which includes county and year effects only), indicate that the two significant positive SDoH predictors of CVD death rate were: percentage of civilian population in armed forces (*p* < 0.01) and median age (*p* < 0.01). The only significant negative SDoH predictor of CVD death rate, was number of federally qualified health centers (*p* < 0.05). Results from the preferred (second) specification were mostly consistent with the first specification, with percentage of civilian population in armed forces (*p* < 0.01) and median age (*p* < 0.05) remaining as significant positive SDoH predictors of CVD death rate, while percentage of population that reported White race (*p* < 0.10) emerged as a significant negative SDoH predictor of CVD death rate.

**Table 2:** The estimated effect of county-level SDoH on the CVD death rate across two specifications

	**Coefficient**	**Robust Standard Error**	**Coefficient**	**Robust Standard Error**
	(1)		(2)	
**Socio-economic context**				
ACS_PCT_FEMALE	0.3409742	0.4329923	0.3979829	0.4319126
ACS_MEDIAN_AGE	1.536069***	0.3724445	0.9442199**	0.4001888
ACS_PCT_BLACK	-0.0107354	0.2413531	0.1794991	0.2397232
ACS_PCT_WHITE	-0.0717861	0.1247179	-0.2094997*	0.1254058
ACS_PCT_HISPAN	0.2419456	0.4314109	0.0770919	0.461046
ACS_PCT_ASIAN	-0.1054069	0.7548355	0.3603354	0.7752729
ACS_PCT_BACHELOR_DGR	-0.3156336	0.2645122	-0.2792628	0.263056
ACS_MEDIAN_HH_INCOME	-0.0000478	0.000145	-0.0002255	0.0001522
ACS_PCT_PERSON_INC99	-0.1662807	0.1749954	-0.1712101	0.1814317
ACS_PCT_UNEMPLOY	-0.0497029	0.2940154	0.0870857	0.3142549
ACS_PCT_GRP_QRT	0.339881	0.2730854	0.3501566	0.2665062
ACS_PCT_VA	-0.4632768	0.3455583	-0.1184386	0.3484473
ACS_PCT_ARMED_FORCES	1.62571***	0.4766393	1.298195***	0.4737176
**Healthcare context**				
AHRF_FED_HLTH_CNT	-0.1742908**	0.0752576	-0.023582	0.0711803
AHRF_RURAL_H_CLINIC	-0.4186592	0.4115517	-0.5452554	0.4158551
**Physical infrastructure context**				
ACS_TOTAL_HOUSEHOLD	0.0000378	0.0000815	-0.0000278	0.0000833
ACS_PCT_RENTED_HH	0.1158973	0.1644035	0.15963	0.1651907
ACS_PCT_MOBILE_HOME	-0.0127164	0.2073848	0.0083726	0.2116054
**County Fixed Effects**	**Yes**		**Yes**	
**Year Fixed Effects**	**Yes**		**Yes**	
**State-by-Year Fixed Effects**	**No**		**Yes**	

Note: *p < 0.10; **p < 0.05; ***p < 0.01. Robust standard errors clustered at the county level

Table 3 shows the impact of significant SDoH predictors (from both specifications), on mean CVD death rate (in the sample), based on both coefficient value and effect size. The approach used to calculate effect size and impact of significant SDoH predictors on mean death rate (i.e., percent change in mean death rate in the sample) is described in Additional file 2 (Supplementary Material). It may be relevant to note that a similar approach, has been leveraged in a vast number of health economic research studies that have utilized similar county-level panel datasets and fixed-effects regression analysis techniques [46–50]. Results from the second specification on Table 3 indicate that 1% increase in population reporting White race is estimated to decrease sample mean CVD death rate by 0.08% based on coefficient value and by 1.40% based on effect size. On the other hand, a 1% increase in civilian population in armed forces is estimated to increase sample mean CVD death rate by 0.52% based on coefficient value and by 0.86% based on effect size. Likewise, a one year increase in median age is estimated to increase sample mean CVD death rate by 0.37% based on coefficient value and by 1.96% based on effect size.

**Table 3:** Impact of significant SDoH predictors on mean CVD death rate

	**Mean**	**S.D**	**Coefficient value (estimated effect on CVD death rate per 100,000)**	**Percent change in mean of DV based on coefficient value**^**a**^	**Effect size per 100,000**^**b**^	**Percent change in mean of DV based on effect size**^**c**^
**Mean of Dependent Variable (CVD Death Rate) in the sample is 250.73 per 100,000**						
**COUNTY & YEAR EFFECTS**						
ACS_MEDIAN_AGE	40.46141	5.203414	1.536069	**0.6126%**	7.99280294	**3.18781%**
ACS_PCT_ARMED_FORCES	0.322018	1.661381	1.62571	**0.6484%**	2.700923706	**1.07722%**
AHRF_FED_HLTH_CNT	1.972197	5.914462	-0.1742908	**-0.0695%**	-1.030836314	**-0.41113%**
**COUNTY, YEAR, & STATE-BY-YEAR FIXED EFFECTS**						
ACS_MEDIAN_AGE	40.46141	5.203414	0.9442199	**0.3766%**	4.913167047	**1.95954%**
ACS_PCT_WHITE	83.3241	16.78994	-0.2094997	**-0.0836%**	-3.517487393	**-1.40290%**
ACS_PCT_ARMED_FORCES	0.322018	1.661381	1.298195	**0.5178%**	2.156796507	**0.86021%**

^**a**^(Coefficient on SDoH IV divided by mean of DV)*100 provides percent change in mean of DV based on coefficient value

^**b**^*Coefficient on SDoH IV multiplied by standard deviation of IV provides effect size of IV per 100,000*

^**c**^(Effect size of SDoH IV divided by mean of DV)*100 provides percent change in mean of DV based on effect size

Table 4 shows two county-level specifications for our second dependent variable of interest, i.e., opioid use death rate. As indicated in the table, under the first specification for county and year fixed effects, the following variables emerged as significant negative SDoH predictors of opioid use death rate: percentage of population that is female (*p* < 0.10) and percentage of civilian population consisting of veterans (*p* < 0.10). The only significant positive SDoH predictor was percentage of population with income to poverty ratio < 1.00 (*p* < 0.01). Results from the second specification for opioid use death rate, indicated greater sensitivity to state-by-year fixed effects compared to CVD death rate. In other words, compared to CVD, there was very little overlap in significant SDoH predictors of opioid use death rate emerging from the two specifications. After adjusting for state-by-year fixed effects, the following four variables emerged as significant negative SDoH predictors of opioid use death rate: median age (*p* < 0.05), percentage of population reporting Hispanic ethnicity (*p* < 0.10); median household income (*p* < 0.10); and percentage of civilian population consisting of veterans (*p* < 0.10).

**Table 4:** The estimated effect of county-level SDoH on the opioid death rate across two specifications

	**Coefficient**	**Robust Standard Error**	**Coefficient**	**Robust Standard Error**
	(1)		(2)	
**Socio-economic context**				
ACS_PCT_FEMALE	-2.97943*	1.520326	-0.1038778	1.38789
ACS_MEDIAN_AGE	-1.561816	1.06467	-1.818271**	0.9173134
ACS_PCT_BLACK	-0.0260737	0.6232132	-0.8427783	0.6334552
ACS_PCT_WHITE	-0.0427271	0.1831953	0.0736518	0.1366791
ACS_PCT_HISPAN	-0.8499315	0.547428	-0.7622937*	0.4591811
ACS_PCT_ASIAN	-0.7129578	0.7763505	-0.3583486	0.700493
ACS_PCT_BACHELOR_DGR	0.828501	0.7093535	-0.0352932	0.6007481
ACS_MEDIAN_HH_INCOME	-0.0002196	0.0002075	-0.0002722*	0.0001616
ACS_PCT_PERSON_INC99	0.9581279***	0.3668685	0.3505055	0.3763996
ACS_PCT_UNEMPLOY	0.2896208	0.4403138	-1.001564	0.6353915
ACS_PCT_GRP_QRT	0.2479495	1.175182	0.3511598	0.9632748
ACS_PCT_VA	-1.593724*	0.8986767	-1.352879*	0.8021658
ACS_PCT_ARMED_FORCES	1.086618	0.6898298	-0.6789627	0.4940171
**Healthcare context**				
AHRF_FED_HLTH_CNT	0.0510922	0.0335136	0.0419603	0.0328061
AHRF_RURAL_H_CLINIC	-0.0076559	0.3444708	0.3126213	0.226519
**Physical infrastructure context**				
ACS_TOTAL_HOUSEHOLD	0.0000531	0.0000475	0.0000125	0.0000367
ACS_PCT_RENTED_HH	-0.1175498	0.3768721	-0.2319311	0.3980847
ACS_PCT_MOBILE_HOME	1.157881*	0.5888022	0.9330352	0.6705346
**County Fixed Effects**	**Yes**		**Yes**	
**Year Fixed Effects**	**Yes**		**Yes**	
**State-by-Year Fixed Effects**	**No**		**Yes**	

Note: *p < 0.10; **p < 0.05; ***p < 0.01. Robust standard errors clustered at the county level

Table 5 shows the impact of significant SDoH predictors on mean opioid use death rate (in the sample), based on both coefficient value and effect size. Results from the second specification on Table 5 indicate that a one-year increase in median age at the county level, is estimated to decrease mean opioid use death rate (in the sample) by 10.20% based on coefficient value, and 53.09% based on effect size. Also, a one-dollar increase in median household income, is estimated to decrease mean opioid death rate by 0.0015% based on coefficient value, and 20.05% based on effect size. Likewise, a 1% increase in population reporting Hispanic ethnicity, is estimated to decrease mean opioid use death rate by 4.28% based on coefficient value and by 81.92% based on effect size; and 1% increase in veterans in the civilian population, is estimated to decrease mean opioid use death rate by 7.59% based on coefficient value and by 23.29% based on effect size.

**Table 5:** Impact of significant SDoH predictors on mean opioid use death rate

	**Mean**	**S.D**	**Coefficient value (estimated effect on opioid use death rate per 100,000)**	**Percent change in mean of DV based on coefficient value**^**a**^	**Effect size per 100,000**^**b**^	**Percent change in mean of DV based on effect size**^**c**^
**Mean of Dependent Variable (Opioid Use Death Rate) in the sample is 17.82 per 100,000**						
**COUNTY & YEAR EFFECTS**						
ACS_PCT_FEMALE	50.03082	2.420218	-2.97943	**-16.7196%**	-7.210870116	**-40.46504%**
ACS_PCT_PERSON_INC99	16.9234	8.297911	0.9581279	**5.3767%**	7.950460041	**44.61538%**
ACS_PCT_VA	10.18986	3.068378	-1.593724	**-8.9435%**	-4.89014766	**-27.44191%**
ACS_PCT_MOBILE_HOME	12.78241	9.492498	1.157881	**6.4976%**	10.99118308	**61.67892%**
**COUNTY, YEAR, & STATE-BY-YEAR FIXED EFFECTS**						
ACS_MEDIAN_AGE	40.46141	5.203414	-1.818271	**-10.2035%**	-9.461216777	**-53.09325%**
ACS_PCT_HISPAN	83.3241	19.15072	-0.7622937	**-4.2777%**	-14.59847321	**-81.92185%**
ACS_MEDIAN_HH_INCOME	46,030.93	13,128.92	-0.0002722	**-0.0015%**	-3.573692024	**-20.05439%**
ACS_PCT_VA	10.18986	3.068378	-1.352879	**-7.5919%**	-4.15114416	**-23.29486%**

^**a**^(Coefficient on SDoH IV divided by mean of DV)*100 provides percent change in mean of DV based on coefficient value

^**b**^*Coefficient on SDoH IV multiplied by standard deviation of IV provides effect size of IV per 100,000*

^**c**^(Effect size of SDoH IV divided by mean of DV)*100 provides percent change in mean of DV based on effect size

## Discussion

### Summary of findings

The descriptive results of this study (summarized in Figs. 1–4), serve to highlight the national trends and regional, state, and county-level disparities in CVD and opioid use death rates. Nationally, while CVD death rates have steadily declined over the ten-year period examined (2009–2018), death from opioid use has increased dramatically over the same period.

The regional analysis revealed interesting patterns. For example, while Region 2 (New Jersey, New York, Puerto Rico, US Virgin Islands) experienced the biggest decline in CVD death rate of 18.6% over the ten-year period, this region also experienced the biggest increase in opioid use death rate of 407.5% over the same period (2009–2018). Similarly, although Region 1 (Connecticut, Maine, Massachusetts, New Hampshire, Rhode Island, Vermont) had the lowest average mean county CVD death rate, this region had the second-highest opioid use death rate. By comparison, Region 9 (Arizona, California, Hawaii, Nevada, American Samoa, Guam, Northern Mariana Islands) and Region 10 (Alaska, Idaho, Oregon, Washington) both had among the lowest mean county CVD death rate and lowest mean county opioid use death rate over the ten-year period. Both these regions also experienced declining trend in opioid use death rate over the ten-year period.

By state, the analysis revealed that Mississippi had the highest average of mean county CVD death rate, while Puerto Rico recorded the lowest average CVD death rates over the ten-year period. On the other hand, while West Virginia had the highest average of mean county opioid use death rate over the ten year period, Hawaii had the lowest. With respect to county-level disparities, despite a relatively lower mean county CVD death rate over the ten-year period, California had among the highest county-level disparities in CVD death rates. On the other hand, Kentucky, New Mexico, and West Virginia, had among highest county-level disparities for opioid use death rates.

Turning to empirical findings related to the relationship between county-level SDoH and mortality from CVD, after adjusting for state-by-year fixed effects, percentage of civilian population in armed forces and median age emerged as significant SDoH positive predictors of CVD death rate, while percentage of population that reported White race emerged as a significant negative SDoH predictor of CVD death rate. it would be relevant to note that none of these results are counterintuitive. They are corroborated by existing evidence from individual-level SDoH studies. For example, several studies have established that the risk of mortality from CVD increases with age [29, 52] and that Whites have a lower risk of mortality from CVD relative to Blacks and other racial/ethnic minorities [30–32]. According to the US Office of Minority Health, Black Americans were 30% more likely to die from heart disease than non-Hispanic whites in 2018. Although Black adults are 40% more likely to have high blood pressure, they are less likely than non-Hispanic whites to have their blood pressure under control. Black women are 60% more likely to have high blood pressure, as compared to non-Hispanic white women [53]. The county-level results related to armed forces are also corroborated by studies that have found higher rates of chronic disease (including coronary heart disease and cancer) among people in armed forces, relative to civilian peers, due to higher rates of smoking, less sleep, and significantly higher levels of physical activity [54–56].

Next, turning to empirical findings related to the relationship between county-level SDoH and mortality from opioid use, the following four significant negative SDoH predictors of opioid use death rate emerged after adjusting for state-by-year fixed effects: median age, percentage of population reporting Hispanic ethnicity, median household income, and percentage of civilian population consisting of veterans. Similar to the results related to CVD deaths, none of the results related to opioid use deaths are counterintuitive, as they have been corroborated by existing evidence from individual-level studies. For example, existing studies have found that people with lower median household income and those living in poverty have a significantly higher risk of mortality from opioid overdose, compared to counterparts with higher median household income [34, 57]. Likewise, the county-level results related to veterans are consistent with several recent (2020) news releases from the US Office of Veterans Affairs (VA), reporting the VA’s success in reducing prescription opioid use in VA patients by 64%, and long-term opioid use by 70% over the past eight years (following an aggressive opioid safety initiative launched in 2012) [58]. Similarly, the county-level results related to age, are also corroborated by individual-level findings [59–61]. Exposure to opioids has been accompanied by rising rates of opioid poisonings and overdoses among adolescents and young adults. For example, a large-scale longitudinal study found a six-fold increase in opioid use disorder among youths 13 to 25 years of age, between 2001 and 2014 [59]. Finally yet importantly, the county-level results related to Hispanics are corroborated by multiple studies over the past two decades that have found that mortality from prescription opioid overdoses was significantly lower among Hispanic individuals, relative non-Hispanic whites [62, 63]. Recent research has linked lower opioid mortality among Hispanics to health care disparities in the US among Hispanic populations with opioid use disorder. For example, while the number of visits for buprenorphine prescriptions increased from 0.04% to 0.36% in the US between 2012 and 2015, White Americans were more likely to receive treatment compared with other ethnic groups, especially Hispanics [64]. Current literature further suggests that the stigma linked to psychiatric illness in the Hispanic culture, creates barriers to care, which in turn may have a role to play in reducing mortality from opioid overdose among Hispanics [62–64].

It would be relevant to note that while results related to significant SDoH predictors of CVD were fairly consistent between the first and second specifications, there was very little overlap in significant SDoH predictors of opioid use death rate emerging from the two specifications. This suggests that results for opioid use death rates were more sensitive to the inclusion of state-by-year fixed effects over county and year fixed effects, which in turn implies that state-level factors, including health and healthcare policies, may have a substantive role to play in influencing mortality from opioid overdoses.

### Implications for policy and practice

Both the descriptive and empirical results of this study carry important implications for policy and practice. The descriptive analysis revealed that Region 1 and Region 2 could benefit from developing comprehensive opioid use prevention strategies at regional and state levels to counter the growing average death rates from opioid use. On the other hand, select states like Mississippi and Louisiana could benefit from population-wide approaches to reduce higher-than-national average county death rates from CVD. Similarly, select states like Kentucky, New Mexico, and West Virginia could benefit from targeted approaches to reducing stark county-level disparities in opioid use death rates.

Empirical results on the relationship between county-level SDoH and CVD death rate, provide insight into county characteristics that may create an inherent advantage or disadvantage from the perspective of risk reduction for CVD mortality, which in turn, could provide direction to communities on the effective design of such efforts. For example, results indicate that counties with younger populations on average, and higher proportions of population reporting White race, may have inherent advantages in being able to contain CVD mortality risk at a population level, compared with counties with higher proportions of people in military service. The county-level results related to race, when combined with the existing evidence of significantly higher risk for CVD mortality among Blacks (relative to Whites), suggest that counties with higher proportion of Blacks may benefit from population-level strategies to reduce CVD risk, including approaches to reduce hypertension, increase screening for blood cholesterol, improve blood pressure management, increase screening for tobacco use, and improve smoking cessation counseling among Black Americans. Likewise, county-level results related to armed forces, suggests that counties with higher proportions of armed forces (in civilian populations) could benefit from implementing a population-based approach to reducing CVD risk among people in military service, through the primary care system. Evidence indicates that people in military service, may be at higher risk for CVD due to less sleep, higher rates of smoking, and a significantly higher levels of physical activity, compared to civilian counterparts. They are also known to face different access challenges. While the vast majority of those in military service have health insurance, they are less likely to have a personal doctor or health care provider [54–57]. Correspondingly, proactive partnerships between insurers and community-based healthcare organizations to modify risk factors in civilian populations with higher proportions of armed forces, could help to address SDoH barriers and reduce the overall risk CVD mortality at the county level.

On the other hand, results on the relationship between county-level SDoH and CVD death rate, suggest that policy-level interventions and partnerships with community organizations to increase household income, could help to reduce the overall risk of opioid death at the county level. One way of doing this, may be to raise the minimum wage in low-income communities with highest risk of opioid overdoses. It is well known that most of the action in regard to raising the minimum wage in the US, takes place at the state or local levels, rather than at the congressional level. From a practical perspective, the $7.25 federal minimum wage is used in only 21 states. In the 29 other states, minimum wages are higher, ranging from $8.65 in Florida to $15 in D.C. In eight of the states with higher-than-federal minimum wages, some cities and counties have adopted local ordinances that provide for even higher rates than their state’s minimum, accelerate schedules for future increases, or both [65]. Prior research has found at least 46 such cities and counties – most of them in the Los Angeles and San Francisco Bay areas of California. Notably, none of the states with the $7.25 federal minimum have higher local minimums [65]. Coincidentally, California which has among the highest minimum wages, also had among the lowest average opioid use death rates over 2009–2018, at 7.07 per 100,000, whereas Kentucky, which is at the federal minimum wage, had among the highest average opioid use death rates at 34.50 per 100,000.

Supplemental to raising the minimum wage, counties may benefit from targeting demand and supply-side interventions towards low-income households with the highest risk of death from opioid overdoses. Examples of demand-side interventions would include providing naloxone to those at greatest risk for opioid overdose, accompanied by education to family and peers on how to recognize and reverse an opioid overdose. Likewise, supply-side interventions would include provision of evidence-based guidelines for opioid prescriptions and naloxone protocol for providers and pharmacies.

Notably, there was little or no overlap between significant (positive or negative) county-level SDoH predictors of CVD death rate and opioid use death rate, especially after adjusting for state-by-year fixed effects. Overall, the differences in county-level SDoH determinants of CVD and opioid use mortality, suggest that counties facing both problems would need to target different populations and SDoH barriers to reduce mortality risk from CVD and opioid use.

### Limitations and future research avenues

A key limitation of this study was the lack of availability of individual (micro)-level data. Only county (macro)-level data were available for analysis. For example, while we knew percent of Hispanic population in county, percent female population in the county and CVD or opioid use death rate in county, the data did not tell us how many females died of CVD or how many of Hispanic ethnicity died of opioid use. This in turn, limited the ability to calculate relative risk, example there was no way of calculating if Blacks at greater risk of death from CVD or opioid use, relative to those of Hispanic race/ethnicity. The absence of individual-level data also limited our ability to use interaction variables, for example, an interaction of percent of female population in county and percent of Hispanic population in county would not provide us with the proportion of Hispanic females in the county. In addition to lack of individual level data, as discussed under ‘variables of interest’ in the Methodology section, there were limitations in county-level data availability across years, which in turn prevented several SDoH variables from being considered for fixed effects panel data analysis. The only way this could have been addressed would have been to perform panel-data analysis for multiple year ranges, which in turn, was beyond the scope of this study. Nevertheless, this would be a productive avenue to explore for future research. Another potential future research avenue would be to obtain individual-level mortality data alongside individual demographic characteristics and risk factors from death certificates at a national level, to facilitate multi-level, i.e., micro (individual) and macro (county or state) level analyses of SDoH predictors of mortality for CVD and opioid use.

## Conclusion

This study sought to examine the relationship between 1) county-level SDoH and ‘CVD deaths’ and 2) county-level SDoH and ‘opioid use deaths,’ in the US from 2009–2018. An additional objective was to describe the distribution of ‘CVD deaths’ and ‘opioid use deaths’ across regions, states, and counties of the United States over a ten-year period (2009–2018). The study found that some regions (e.g., Region 1, Region 2) that have had success in reducing CVD mortality over the ten-year period have also experienced dramatic increases in opioid use death rates during the same period. The study also found that county-level disparities (within states) for opioid use deaths were significantly higher than county-level disparities for CVD mortality over the ten-year period. For example, in the state of Mississippi, the county with highest CVD rate had a rate that was 183% higher than the county with the lowest CVD rate. On the other hand, in the state of Kentucky, the county with highest opioid use rate had a rate that was 1,360% higher than the county with lowest opioid use rate.

With respect to SDoH, after adjusting for state-by-year fixed effects, percentage of civilian population in armed forces and median age emerged as significant SDoH positive predictors of CVD death rate, while percentage of population that reported White race emerged as a significant negative SDoH predictor of CVD death rate. On the other hand, after adjusting for state-by-year fixed effects, there were four significant negative SDoH predictors of opioid use death rate, including: median age, percentage of population reporting Hispanic ethnicity, median household income, and percentage of civilian population consisting of veterans. There was little to no overlap between significant county-level SDoH predictors of CVD and opioid use death rates, indicating that counties facing both significant public health challenges, would need to address different populations and SDoH barriers for tackling both problems. Additionally, in both areas (CVD and opioid use), none of the county-level SDoH results are counterintuitive, i.e., they are corroborated by existing evidence from individual-level studies.

The study provides several implications for policy, practice, and future research for addressing SDoH barriers to reduce risk for CVD mortality in (counties) communities, including proactive partnerships between insurers and healthcare organizations in populations with higher proportions of armed forces, to modify specific risk factors for CVD mortality in this population. Likewise, strategies to address SDoH barriers to reduce risk of opioid use mortality include, increasing household income by raising county-level minimum wage in low-income high-risk communities, coupled with demand-side interventions (e.g., improving access to treatment services) and supply-side interventions (e.g., provision of evidence-based guidelines for opioid prescriptions in the emergency and outpatient settings), to reduce the risk of mortality from opioid overuse in these communities.

## Supplementary Information

**Additional file 1.**

**Additional file 2.**

## Data Availability

The data analyzed in this study is a publicly available secondary database that can be freely downloaded from the link below. https://www.ahrq.gov/sdoh/data-analytics/sdoh-data.html

## Abbreviations

SDoH: Social Determinants of Health
CVD: Cardiovascular Disease
CDC: Centers for Disease Control and Prevention
AHA: American Heart Association
ACS: American Community Survey
NCHS: National Center for Health Statistics
US: United States
VA: Veterans Affairs
ICD-10: International Classification of Diseases, Tenth Revision
MCOD: Multiple Cause of Death Codes
