# Supplementary material for: The relationship between Social Determinants of Health (SDoH) and death from cardiovascular disease or opioid use in counties across the United States (2009–2018)
Source: BMC Public Health. 2022 Feb 4;22:236. doi: 10.1186/s12889-022-12653-8 (PMC8817535; doi:10.1186/s12889-022-12653-8)
Supplement: Supplementary file 1 — Additional file 1. [file 12889_2022_12653_MOESM1_ESM.pdf]

Appendix 1

SDoH variables in AHRQ Dataset and Rationale for Exclusion/Inclusion

Variable Label	Variable description	Two Criteria for Exclusion		Excluded or Included?
		Problem with 10-year Data Availability?	Problem with Multi-Collinearity?	
**Socio-economic context**				
ACS_PCT_FEMALE	Percentage of population that is female	No	No	Included
ACS_MEDIAN_AGE	Median Age	No	No	Included
ACS_PCT_ASIAN	Percentage of population reporting Asian race	No	No	Included
ACS_PCT_BLACK	Percentage of population reporting Black race	No	No	Included
ACS_PCT_HISPAN	Percentage of population reporting Hispanic ethnicity	No	No	Included
ACS_PCT_WHITE	Percentage of population reporting White race	No	No	Included
ACS_PCT_BACHELOR_DGR	Percentage of population with a bachelor's degree (ages 25 and over)	No	No	Included
ACS_MEDIAN_HH_INCOME	Median household income	No	No	Included
ACS_PCT_INC137	Percentage of population with income to poverty ratio of 1.00–1.37	Yes	No	Excluded
ACS_PCT_INC400	Percentage of population with income to poverty ratio of 4.00 or higher	Yes	No	Excluded
ACS_PCT_UNEMPLOY	Percentage of population that was unemployed (ages 16 years and over)	No	No	Included
ACS_PCT_CONSTRUCT	Percentage of employed working in construction	Yes	No	Excluded
ACS_PCT_MANUFACT	Percentage of employed working in manufacturing	Yes	No	Excluded
ACS_PCT_NATURE	Percentage of employed working in agriculture, forestry, fishing and	Yes	No	Excluded
ACS_PCT_PROFESS	Percentage of employed working in professional, scientific, management,	Yes	No	Excluded
ACS_PCT_GRP_QRT	Percentage of persons in institutionalized (correctional) group	No	No	Included
ACS_PCT_VA	Percentage of the civilian population consisting of veterans (ages 18 and	No	No	Included
ACS_PCT_ARMED_FORCES	Percentage of civilian population in armed forces (ages 16 years and over)	No	No	Included
ACS_PCT_DISABLE	Percentage of population with a disability	Yes	No	Excluded
**Healthcare Context**				
AHRF_HPSA_PRIM	HPSA code—shortage of primary care physicians	Yes	No	Excluded
AHRF_HPSA_MENTAL	HPSA code—shortage of mental healthcare providers	Yes	No	Excluded
AMFAR_AMATFAC	Number of substance abuse treatment facilities with all three	Yes	No	Excluded

Variable Label	Variable description	Two Criteria for Exclusion		Excluded or Included?
		Problem with 10-year Data Availability?	Problem with Multi-Collinearity?	
AMFAR_HICVTFAC	Number of substance abuse facilities offering both HIV test and hepatitis C	Yes	No	Excluded
AMFAR_HIVTFAC	Number of substance abuse facilities offering HIV testing	Yes	No	Excluded
AMFAR_MHFAC	Number of facilities that provide mental health services	Yes	No	Excluded
ACS_PCT_MEDICAID_ANY	Percentage of population with any Medicaid/means-tested public health	Yes	No	Excluded
ACS_PCT_MEDICARE_ONLY	Percentage of population with Medicare only	Yes	No	Excluded
ACS_PCT_PRIVATE_ANY	Percentage of population with any private health insurance coverage	Yes	No	Excluded
ACS_PCT_PRIVATE_EMPL	Percentage of population with employer-based health insurance	Yes	No	Excluded
ACS_PCT_PUBLIC_ONLY	Percentage of population with Medicare, Medicaid,	Yes	No	Excluded
ACS_PCT_TRICARE_VA	Percentage of population with TRICARE/military or VA health	Yes	No	Excluded
ACS_PCT_UNINSURED	Percentage of population with no health insurance coverage	Yes	No	Excluded
AMFAR_OPIOIDRXRATE	Number of opioid prescriptions per 100 persons	Yes	No	Excluded
CHR_ADULT_OBESITY	Percentage of the adult population (age 20 and older) that is obese	Yes	No	Excluded
CHR_EXCESS_DRINK	Percentage of adults reporting binge or heavy drinking	Yes	No	Excluded
CHR_SMOKING	Percentage of adults who are current smokers	Yes	No	Excluded
CHR_DIABETES_PREV	Percentage of adults aged 20 and above with diagnosed diabetes	Yes	No	Excluded
CHR_MENTAL_DISTRESS	Percentage of adults reporting 14 or more days of poor mental health per	Yes	No	Excluded
CHR_PHYSICAL_DISTRESS	Percentage of adults reporting 14 or more days of poor physical health per	Yes	No	Excluded
CHR_PHYS_INACTIVITY	Percentage of adults age 20 and over reporting no leisure-time physical	Yes	No	Excluded
CHR_TEEN_BIRTH	Births per 1,000 females aged 15-19	Yes	No	Excluded
CHR_SEGREG_BLACK	Segregation Index (higher values indicate greater residential	Yes	No	Excluded
CHR_SEGREG_NON_WHITE	Segregation Index (higher values indicate greater residential	Yes	No	Excluded
AHRF_ER_VST_ST_G_HOSP	Number of emergency department visits in short-term general hospitals	Yes	No	Excluded
AHRF_HOSP_AMMS	Total number of hospital admissions	Yes	No	Excluded
AHRF_ST_COMM_HOSP_ADMS	Total number of short-term community hospital admissions	Yes	No	Excluded
AHRF_MCR_IP_DAY_ST_G	Total Medicare inpatient days, short-term general hospitals	Yes	No	Excluded
AHRF_MDCD_INP_DAY_STGH	Total Medicaid inpatient days, short-term general hospitals	Yes	No	Excluded
AHRF_IP_DAY_ST_G_HOSP	Total number of inpatient days in short-term general hospitals	Yes	No	Excluded

Variable Label	Variable description	Two Criteria for Exclusion		Excluded or Included?
		Problem with 10-year Data Availability?	Problem with Multi-Collinearity?	
AHRF_RATE_MCR_BN_READM	Medicare beneficiary hospital readmission rate, fee for service	Yes	No	Excluded
AHRF_OP_VST_ST_G_ER_OP	Total number of outpatient visits in short-term general hospital	Yes	No	Excluded
AHRF_MDCR_FFS_STD_COST	Total standardized Medicare costs, fee for service	Yes	No	Excluded
AHRF_N_STGH_EXP_1000	Total expenses (in $1000s) from facilities reporting expenses	Yes	No	Excluded
CHR_MENTAL_DR_RATE	Number of mental health care providers per 100,000 population	Yes	No	Excluded
CHR_MENTAL_DR_NUM	Number of mental health care providers (numerator)	Yes	No	Excluded
AHRF_TOT_HOSPS	Total number of hospitals	Yes	No	Excluded
AHRF_COMM_HLTH_CNTR	Number of community health centers, grantees only	Yes	No	Excluded
AHRF_FED_HLTH_CNT	Number of Federally Qualified Health Centers	No	No	Included
AHRF_RURL_REFRRL_CNT	Total number of rural referral centers	Yes	No	Excluded
AHRF_RURAL_H_CLINIC	Number of rural health clinics	No	No	Included
AHRF_CARDIOVAS_DIS	Total number of cardiovascular disease specialists	Yes	No	Excluded
AHRF_MED_SPEC	Total number of medical specialists	Yes	No	Excluded
AHRF_ER_MED	Total number of non-federal emergency medicine physicians	Yes	No	Excluded
AHRF_GEN_INTERNAL_MED	Total number of non-federal general internal medicine physicians	Yes	No	Excluded
AHRF_PHYS_PRIMARY	Total number of non-federal primary care physicians	Yes	No	Excluded
AHRF_TOTAL_MDS	Total number of non-federal MDs	Yes	No	Excluded
CHR_PRIMARY_DR_RATE	Number of primary care physicians per 100,000 population	Yes	No	Excluded
CHR_PRIMARY_DR_NUM	Number of primary care physicians (numerator)	Yes	No	Excluded
**Physical Infrastructure Context**				
ACS_TOTAL_HOUSEHOLD	Total number of households	No	No	Included
ACS_PCT_RENTED_HH	Percentage of occupied housing units: rented	No	No	Included
ACS_PCT_10UNITS	Percentage of housing in structures with 10 or more units	Yes	No	Excluded
ACS_PCT_HH_PUB_ASSIST	Percentage households with public assistance income or food	Yes	No	Excluded
ACS_PCT_MOBILE_HOME	Percentage of housing units that are mobile homes	No	No	Included
ACS_PCT_PUBL_TRANSIT	Percentage of workers taking public transportation, excluding taxicab	Yes	No	Excluded
CHR_HOMICIDES	Deaths due to homicide per 100,000 population	Yes	No	Excluded

Variable Label	Variable description	Two Criteria for Exclusion		Excluded or Included?
		**Problem with 10-year Data Availability?**	**Problem with Multi-Collinearity?**	
CHR_FIREARM_DEATH	Deaths due to firearms per 100,000 population	Yes	No	Excluded
CHR_VIOLENT_CRIME	Reported violent crime offenses per 100,000 population	Yes	No	Excluded
AHRF_USDA_RUCC_2013	2013 Rural-Urban Continuum Code (USDA ERS)	No	Yes	Excluded
CEN_AREALAND_SQM	Land areas in square miles	No	Yes	Excluded
CEN_POPDENSITY	Population density	Yes	Yes	Excluded
CCBP_RATE_BWLSTORES_PER_1000	Beer, wine and liquor stores per 1,000 people	No	Yes	Excluded
CCBP_RATE_SOGS_PER_1000	Supermarkets and other grocery (except convenience) stores per 1,000	No	Yes	Excluded
CCBP_RATE_FF_PER_1000	Limited service restaurants (fast food establishments) per 1,000 people	No	Yes	Excluded
CCBP_RATE_FSR_PER_1000	Full service restaurants per 1,000 people	No	Yes	Excluded
CCBP_RATE_CS_PER_1000	Convenience stores per 1,000 people	No	Yes	Excluded
CCBP_RATE_SFS_PER_1000	Specialized food stores per 1,000 people	No	Yes	Excluded
CCBP_RATE_CFS_PER_1000	Community food services (targeting low-income or elderly) per 1,000	No	Yes	Excluded
CCBP_RATE_FCRSC_PER_1000	Fitness centers and recreational sports centers per 1,000 people	No	Yes	Excluded
CCBP_RATE_GAMBLING_PER_1000	Gambling establishments per 1,000 people	No	Yes	Excluded
CCBP_RATE_CHS_PER_1000	Community housing services (targeting low-income or elderly) per	No	Yes	Excluded
CCBP_RATE_CFHEORS_PER_1000	Emergency and other relief services for victims of domestic or	No	Yes	Excluded
CCBP_RATE_SHELTERS_PER_1000	Temporary shelters per 1,000 people	No	Yes	Excluded
AHRF_PCT_GOOD_AQ	Percentage of days with good air quality	Yes	No	Excluded
NEPHTN_MODAAC_PM25	Annual average ambient concentrations of PM2.5 in	Yes	No	Excluded
NEPHTN_HEATIND_100	Extreme heat - 100°F: Number of days with daily maximum heat index,	Yes	No	Excluded
NEPHTN_HEATIND_105	Extreme heat - 105°F: Number of days with daily maximum heat index,	Yes	No	Excluded
NEPHTN_HEATIND_90	Extreme heat - 90°F: Number of days with daily maximum index, absolute	Yes	No	Excluded
NEPHTN_HEATIND_95	Extreme heat - 95°F: Number of days with daily maximum heat index,	Yes	No	Excluded
NEPHTN_LEAD	Percentage of children tested with confirmed blood lead levels 10 µg/dL	Yes	No	Excluded
NEPHTN_NUMDROUGHT	Number of months of mild drought or worse per year	Yes	No	Excluded
NEPHTN_TEMPERATURE_100	Extreme heat - 100°F: Number of days with daily maximum temperature,	Yes	No	Excluded
NEPHTN_TEMPERATURE_105	Extreme heat - 105°F: Number of days with daily maximum temperature,	Yes	No	Excluded

NEPHTN_TEMPERATURE_90	Extreme heat - 90°F: Number of days with daily maximum temperature,	Yes	No	Excluded
NEPHTN_TEMPERATURE_95	Extreme heat - 95°F: Number of days with daily maximum temperature,	Yes	No	Excluded
