# Supplementary material for: The relationship between Social Determinants of Health (SDoH) and death from cardiovascular disease or opioid use in counties across the United States (2009–2018)
Source: BMC Public Health. 2022 Feb 4;22:236. doi: 10.1186/s12889-022-12653-8 (PMC8817535; doi:10.1186/s12889-022-12653-8)
Supplement: Supplementary file 2 — Additional file 2. [file 12889_2022_12653_MOESM2_ESM.pdf]

APPENDIX 2

STATISTICAL SUPPLEMENT

It would be relevant to note that our preferred fixed effects models indicated moderate to high correlation between fixed effects (or unobserved heterogeneity) and our independent variables, implying that our models did well by controlling for these fixed effects. The fixed effects models that included county and year fixed-effects only (i.e., 'Specification 1' for both CVD and opioid use death rates), were each generated using two approaches, 1) the 'fixed effects estimator' approach and 2) the 'least square dummy variables' approach, to ensure that the estimates (regression coefficient values) generated by both approaches, matched each other. The results revealed an exact match in coefficient values from both approaches for both CVD and opioid use death rates. Additionally, the model R-square in the 'least squares dummy variables' models were 0.8776 for CVD mortality and 0.8392 for opioid use mortality, indicating that these models served to explain a large portion of the variance in CVD mortality and opioid use mortality, respectively.

CONTENTS OF THIS SUPPLEMENTARY FILE

1. **STATA OUTPUT - OPIOID USE DEATH RATE**

   Page 2. COUNTY AND YEAR FIXED-EFFECTS FOR OPIOID USE DEATH RATE
   - Table 4, Specification 1 (in manuscript)

   Page 3. COUNTY, YEAR, and STATE-BY-YEAR FIXED-EFFECTS FOR OPIOID USE DEATH RATE
   - Table 4, Specification 2 (in manuscript)

2. **STATA OUTPUT - CVD DEATH RATE**

   Page 10. COUNTY AND YEAR FIXED-EFFECTS FOR CVD DEATH RATE
   - Table 2, Specification 1 (in manuscript)

   Page 11. COUNTY, YEAR, and STATE-BY-YEAR FIXED-EFFECTS FOR CVD DEATH RATE
   - Table 2, Specification 2 (in manuscript)

3. **TOTAL NUMBER OF MISSING OBSERVATIONS BY DEPENDENT VARIABLE**

   Page 19

4. **METHOD FOR CALCULATING IMPACT OF SIGNIFICANT PREDICTORS ON SAMPLE MEAN MORTALITY RATES (TABLES 3 AND TABLE 5)**

   Page 20

COUNTY AND YEAR FIXED EFFECTS FOR OPIOID USE DEATH RATE (TABLE 4, SPECIFICATION 1)

```
Fixed-effects (within) regression               Number of obs     =      3,272
Group variable: county_fip~e                    Number of groups  =        554

R-sq:                                           Obs per group:
     within  = 0.3659                                         min =          1
     between = 0.1756                                         avg =        5.9
     overall = 0.1476                                         max =         10

                                                F(28,553)         =      17.30
corr(u_i, Xb)  = -0.9434                        Prob > F          =     0.0000

                              (Std. Err. adjusted for 554 clusters in county_fipscode)
--------------------------------------------------------------------------------------
                     |               Robust
    cdc_opioid_death |      Coef.   Std. Err.      t    P>|t|     [95% Conf. Interval]
---------------------+----------------------------------------------------------------
      acs_pct_female |   -2.97943   1.520326    -1.96   0.051    -5.965751    .0068906
      acs_median_age |  -1.561816    1.06467    -1.47   0.143    -3.653108    .5294754
       acs_pct_black |  -.0260737   .6232132    -0.04   0.967    -1.250228    1.198081
       acs_pct_white |  -.0427271   .1831953    -0.23   0.816    -.4025709    .3171167
      acs_pct_hispan |  -.8499315    .547428    -1.55   0.121    -1.925224    .2253611
       acs_pct_asian |  -.7129578   .7763505    -0.92   0.359    -2.237914    .8119988
acs_pct_bachelor_dgr |    .828501   .7093535     1.17   0.243    -.5648559    2.221858
acs_median_hh_income |  -.0002196   .0002075    -1.06   0.290    -.0006272     .000188
acs_pct_person_inc99 |   .9581279   .3668685     2.61   0.009     .2375016    1.678754
    acs_pct_unemploy |   .2896208   .4403138     0.66   0.511    -.5752713    1.154513
     acs_pct_grp_qrt |   .2479495   1.175182     0.21   0.833    -2.060416    2.556315
          acs_pct_va |  -1.593724   .8986767    -1.77   0.077    -3.358962    .1715133
acs_pct_armed_forces |   1.086618   .6898298     1.58   0.116    -.2683886    2.441626
   ahrf_fed_hlth_cnt |   .0510922   .0335136     1.52   0.128    -.0147372    .1169217
 ahrf_rural_h_clinic |  -.0076559   .3444708    -0.02   0.982    -.6842871    .6689752
 acs_total_household |   .0000531   .0000475     1.12   0.264    -.0000401    .0001464
   acs_pct_rented_hh |  -.1175498   .3768721    -0.31   0.755    -.8578258    .6227261
 acs_pct_mobile_home |   1.157881   .5888022     1.97   0.050      .001319    2.314444
    acs_total_pop_wt |  -.0000605   .0000127    -4.75   0.000    -.0000855   -.0000355
                     |
                year |
               2010  |   -.603591   1.605359    -0.38   0.707    -3.756938    2.549756
               2011  |   1.100544   1.486412     0.74   0.459    -1.819161    4.020249
               2012  |   .9990104   1.915128     0.52   0.602    -2.762806    4.760826
               2013  |   1.441987   2.373825     0.61   0.544     -3.22083    6.104803
               2014  |   3.318097   2.868838     1.16   0.248    -2.317054    8.953249
               2015  |   6.083412   3.384051     1.80   0.073    -.5637549    12.73058
               2016  |   11.08971   3.977029     2.79   0.005     3.277778    18.90164
               2017  |   14.55504   4.715348     3.09   0.002     5.292857    23.81723
               2018  |   14.81599   5.323405     2.78   0.006     4.359425    25.27256
                     |
               _cons |    257.825   97.31878     2.65   0.008     66.66534    448.9847
---------------------+----------------------------------------------------------------
             sigma_u |  33.304578
             sigma_e |  6.7348966
                 rho |   .9607131   (fraction of variance due to u_i)
--------------------------------------------------------------------------------------
```

COUNTY, YEAR, & STATE-BY-YEAR FIXED EFFECTS FOR OPIOID USE DEATH RATE (TABLE 4, SPECIFICATION 2)

```
Fixed-effects (within) regression               Number of obs     =      3,272
Group variable: county_fip~e                    Number of groups  =        554

R-sq:                                           Obs per group:
     within  = 0.6004                                         min =          1
     between = 0.2305                                         avg =        5.9
     overall = 0.2090                                         max =         10

                                                F(271,553)        =    2175.00
corr(u_i, Xb)  = -0.7433                        Prob > F          =     0.0000

                              (Std. Err. adjusted for 554 clusters in county_fipscode)
```

cdc_opioid_death	Coef.	Robust Std. Err.	t	P>|t|	[95% Conf.	Interval]
acs_pct_female	-.1038778	1.38789	-0.07	0.940	-2.830058	2.622302
acs_median_age	-1.818271	.9173134	-1.98	0.048	-3.620116	-.0164267
acs_pct_black	-.8427783	.6334552	-1.33	0.184	-2.087051	.4014943
acs_pct_white	.0736518	.1366791	0.54	0.590	-.1948218	.3421255
acs_pct_hispan	-.7622937	.4591811	-1.66	0.097	-1.664246	.1396588
acs_pct_asian	-.3583486	.700493	-0.51	0.609	-1.734301	1.017604
acs_pct_bachelor_dgr	-.0352932	.6007481	-0.06	0.953	-1.215321	1.144734
acs_median_hh_income	-.0002722	.0001616	-1.69	0.093	-.0005896	.0000451
acs_pct_person_inc99	.3505055	.3763996	0.93	0.352	-.3888424	1.089853
acs_pct_unemploy	-1.001564	.6353915	-1.58	0.116	-2.24964	.2465124
acs_pct_grp_qrt	.3511598	.9632748	0.36	0.716	-1.540965	2.243285
acs_pct_va	-1.352879	.8021658	-1.69	0.092	-2.928544	.2227854
acs_pct_armed_forces	-.6789627	.4940171	-1.37	0.170	-1.649342	.2914168
ahrf_fed_hlth_cnt	.0419603	.0328061	1.28	0.201	-.0224796	.1064001
ahrf_rural_h_clinic	.3126213	.226519	1.38	0.168	-.1323216	.7575642
acs_total_household	.0000125	.0000367	0.34	0.733	-.0000596	.0000846
acs_pct_rented_hh	-.2319311	.3980847	-0.58	0.560	-1.013874	.550012
acs_pct_mobile_home	.9330352	.6705346	1.39	0.165	-.3840712	2.250141
acs_total_pop_wt	-9.70e-06	.000011	-0.88	0.377	-.0000312	.0000118
year						
2010	5.816762	2.386956	2.44	0.015	1.128153	10.50537
2011	4.764055	2.174633	2.19	0.029	.4925033	9.035606
2012	5.686462	2.470515	2.30	0.022	.8337208	10.5392
2013	8.585415	2.850783	3.01	0.003	2.985728	14.1851
2014	9.82001	3.363072	2.92	0.004	3.214051	16.42597
2015	7.878705	3.585629	2.20	0.028	.8355862	14.92182
2016	12.17845	4.022629	3.03	0.003	4.276944	20.07995
2017	15.72429	4.360762	3.61	0.000	7.158604	24.28997
2018	12.78837	5.006174	2.55	0.011	2.954926	22.62181
state_year_num						
Alabama2010	-6.646825	2.435963	-2.73	0.007	-11.4317	-1.861953
Alabama2011	-8.900078	2.893243	-3.08	0.002	-14.58317	-3.216988
Alabama2012	-7.889398	2.527957	-3.12	0.002	-12.85497	-2.923826
Alabama2013	-5.45175	1.804021	-3.02	0.003	-8.995323	-1.908177
Alabama2014	1.781881	2.785022	0.64	0.523	-3.688634	7.252397
Alabama2015	1.672318	3.702419	0.45	0.652	-5.600206	8.944842
Alabama2016	-1.609039	5.311465	-0.30	0.762	-12.04215	8.824075
Alabama2017	-3.79635	4.448594	-0.85	0.394	-12.53456	4.941858
Alabama2018	-2.212069	4.182585	-0.53	0.597	-10.42777	6.003628
Alaska2009	14.56081	3.979109	3.66	0.000	6.744795	22.37683
Alaska2010	8.192934	4.190749	1.96	0.051	-.0387986	16.42467
Alaska2011	8.871232	3.252741	2.73	0.007	2.481993	15.26047
Alaska2012	8.346118	2.950923	2.83	0.005	2.549729	14.14251
Alaska2013	5.825659	2.777884	2.10	0.036	.3691634	11.28216
Alaska2014	4.459551	3.081679	1.45	0.148	-1.593677	10.51278
Alaska2015	6.002479	2.552617	2.35	0.019	.9884671	11.01649
Alaska2016	3.555525	1.360745	2.61	0.009	.8826635	6.228386
Alaska2017	4.825554	2.142893	2.25	0.025	.6163479	9.03476
Alaska2018	0	(omitted)				
Arizona2009	5.313043	4.4236	1.20	0.230	-3.37607	14.00216
Arizona2010	4.167429	5.404506	0.77	0.441	-6.448443	14.7833

Arizona2011	3.47981	4.736037	0.73	0.463	-5.823013	12.78263
Arizona2012	3.085829	4.60559	0.67	0.503	-5.960762	12.13242
Arizona2013	.8983563	4.402184	0.20	0.838	-7.748692	9.545404
Arizona2014	-.776917	4.13928	-0.19	0.851	-8.907552	7.353718
Arizona2015	3.613812	4.464939	0.81	0.419	-5.156503	12.38413
Arizona2016	-1.156542	2.448467	-0.47	0.637	-5.965976	3.652892
Arizona2017	-4.592151	2.53194	-1.81	0.070	-9.565547	.3812442
Arizona2018	0	(omitted)				
Arkansas2009	3.954104	3.158678	1.25	0.211	-2.25037	10.15858
Arkansas2010	5.596005	3.512223	1.59	0.112	-1.302925	12.49493
Arkansas2011	2.280555	3.351184	0.68	0.496	-4.302051	8.863162
Arkansas2012	.3150828	3.272783	0.10	0.923	-6.113525	6.74369
Arkansas2013	-.0074553	3.02345	-0.00	0.998	-5.946306	5.931395
Arkansas2014	-1.035484	3.605304	-0.29	0.774	-8.11725	6.046283
Arkansas2015	-.4801002	3.579416	-0.13	0.893	-7.511015	6.550814
Arkansas2016	-4.340978	2.969062	-1.46	0.144	-10.173	1.491042
Arkansas2017	-6.749472	2.73499	-2.47	0.014	-12.12171	-1.377231
Arkansas2018	0	(omitted)				
California2009	6.829243	2.826822	2.42	0.016	1.276622	12.38186
California2010	5.414934	3.173386	1.71	0.089	-.8184297	11.6483
California2011	4.730443	2.870286	1.65	0.100	-.907554	10.36844
California2012	3.440495	2.742468	1.25	0.210	-1.946434	8.827424
California2013	1.288867	2.499434	0.52	0.606	-3.620679	6.198412
California2014	1.006741	2.917278	0.35	0.730	-4.723559	6.737042
California2015	2.214998	2.420271	0.92	0.360	-2.539051	6.969048
California2016	-2.094621	1.098709	-1.91	0.057	-4.252775	.0635326
California2017	-4.702649	2.025192	-2.32	0.021	-8.680659	-.7246395
California2018	0	(omitted)				
Colorado2009	7.45438	3.714101	2.01	0.045	.1589084	14.74985
Colorado2010	3.055567	3.591661	0.85	0.395	-3.9994	10.11053
Colorado2011	4.558344	3.295294	1.38	0.167	-1.91448	11.03117
Colorado2012	4.887616	2.898005	1.69	0.092	-.8048285	10.58006
Colorado2013	2.675532	2.954198	0.91	0.366	-3.12729	8.478353
Colorado2014	2.531451	2.951871	0.86	0.391	-3.266799	8.329702
Colorado2015	2.097725	2.57309	0.82	0.415	-2.9565	7.151951
Colorado2016	-1.546532	.9446696	-1.64	0.102	-3.402111	.3090478
Colorado2017	-3.934285	2.146209	-1.83	0.067	-8.150004	.2814335
Colorado2018	0	(omitted)				
Connecticut2009	-15.06519	3.722312	-4.05	0.000	-22.37679	-7.753586
Connecticut2010	-16.18508	3.965099	-4.08	0.000	-23.97357	-8.396578
Connecticut2011	-16.89791	3.88954	-4.34	0.000	-24.53799	-9.257832
Connecticut2012	-16.35691	3.540344	-4.62	0.000	-23.31107	-9.402739
Connecticut2013	-13.00978	3.853014	-3.38	0.001	-20.57811	-5.441445
Connecticut2014	-10.60894	3.51173	-3.02	0.003	-17.5069	-3.710981
Connecticut2015	-7.954282	3.56702	-2.23	0.026	-14.96085	-.9477172
Connecticut2016	-5.982237	3.170503	-1.89	0.060	-12.20994	.2454656
Connecticut2017	-4.164874	3.253725	-1.28	0.201	-10.55605	2.226298
Connecticut2018	0	(omitted)				
Delaware2009	-24.27757	3.717463	-6.53	0.000	-31.57964	-16.97549
Delaware2010	-23.14182	3.983893	-5.81	0.000	-30.96724	-15.31641
Delaware2011	-21.08409	4.403089	-4.79	0.000	-29.73291	-12.43526
Delaware2012	-21.56666	5.217795	-4.13	0.000	-31.81579	-11.31754
Delaware2013	-22.2573	5.009942	-4.44	0.000	-32.09815	-12.41646
Delaware2014	-21.44856	6.22195	-3.45	0.001	-33.6701	-9.227011
Delaware2015	-19.59698	4.33149	-4.52	0.000	-28.10516	-11.08879
Delaware2016	-25.80849	4.758109	-5.42	0.000	-35.15467	-16.46232
Delaware2017	-17.36592	2.226038	-7.80	0.000	-21.73845	-12.9934
Delaware2018	0	(omitted)				
Dist. of Columbia2010	-10.71787	5.106113	-2.10	0.036	-20.74762	-.6881237
Dist. of Columbia2011	-10.79532	4.395932	-2.46	0.014	-19.43009	-2.160555
Dist. of Columbia2012	-12.75646	3.919897	-3.25	0.001	-20.45617	-5.056753
Dist. of Columbia2013	-14.46331	3.2016	-4.52	0.000	-20.75209	-8.174522
Dist. of Columbia2014	-14.78457	3.317263	-4.46	0.000	-21.30055	-8.268596
Dist. of Columbia2015	-8.395736	2.694578	-3.12	0.002	-13.6886	-3.102876
Dist. of Columbia2016	2.668813	1.317039	2.03	0.043	.0818014	5.255824
Dist. of Columbia2017	4.549055	2.013657	2.26	0.024	.5937028	8.504408
Dist. of Columbia2018	0	(omitted)				
Florida2009	-2.528809	3.421146	-0.74	0.460	-9.248839	4.191221
Florida2010	-.7846643	3.698923	-0.21	0.832	-8.050322	6.480993
Florida2011	-3.032281	3.391231	-0.89	0.372	-9.693551	3.628988
Florida2012	-6.016637	3.213631	-1.87	0.062	-12.32905	.2957795
Florida2013	-8.480415	2.850152	-2.98	0.003	-14.07886	-2.881967
Florida2014	-7.8471	3.275938	-2.40	0.017	-14.2819	-1.412295
Florida2015	-2.161008	3.074491	-0.70	0.482	-8.200118	3.878101
Florida2016	-3.140567	2.395265	-1.31	0.190	-7.845497	1.564362
Florida2017	-5.932715	2.563947	-2.31	0.021	-10.96898	-.8964489

Florida2018	|	0	(omitted)				
Georgia2009	|	-2.149265	4.567887	-0.47	0.638	-11.1218	6.823267
Georgia2010	|	.1697815	3.40599	0.05	0.960	-6.520479	6.860042
Georgia2011	|	.7121356	3.325918	0.21	0.831	-5.820842	7.245113
Georgia2012	|	.4357548	3.12849	0.14	0.889	-5.709423	6.580932
Georgia2013	|	-.9693001	2.586314	-0.37	0.708	-6.0495	4.1109
Georgia2014	|	-1.796175	3.258633	-0.55	0.582	-8.196987	4.604637
Georgia2015	|	2.346649	2.604675	0.90	0.368	-2.769617	7.462915
Georgia2016	|	-2.090992	1.374402	-1.52	0.129	-4.790679	.608696
Georgia2017	|	-5.786419	2.339683	-2.47	0.014	-10.38217	-1.190666
Georgia2018	|	0	(omitted)				
Hawaii2009	|	11.83703	3.717101	3.18	0.002	4.53566	19.13839
Hawaii2010	|	12.19558	4.115502	2.96	0.003	4.111652	20.27951
Hawaii2011	|	9.23636	3.408234	2.71	0.007	2.541692	15.93103
Hawaii2012	|	7.79297	3.158657	2.47	0.014	1.588538	13.9974
Hawaii2013	|	5.510083	2.718769	2.03	0.043	.1697056	10.85046
Hawaii2014	|	2.514484	3.023782	0.83	0.406	-3.425019	8.453988
Hawaii2015	|	5.019826	2.498331	2.01	0.045	.1124478	9.927204
Hawaii2016	|	1.755725	1.099261	1.60	0.111	-.4035128	3.914963
Hawaii2017	|	-2.856451	1.991929	-1.43	0.152	-6.769124	1.056221
Hawaii2018	|	0	(omitted)				
Idaho2009	|	7.395892	2.358986	3.14	0.002	2.762223	12.02956
Idaho2010	|	3.71809	2.8156	1.32	0.187	-1.812489	9.248669
Idaho2011	|	3.009771	2.593214	1.16	0.246	-2.083983	8.103524
Idaho2013	|	-.026366	2.286062	-0.01	0.991	-4.516792	4.46406
Idaho2014	|	-.5583259	2.773988	-0.20	0.841	-6.007167	4.890515
Idaho2015	|	-1.712617	2.355101	-0.73	0.467	-6.338655	2.913422
Idaho2016	|	-4.663594	.836064	-5.58	0.000	-6.305843	-3.021344
Idaho2017	|	-7.428335	1.930937	-3.85	0.000	-11.2212	-3.635466
Idaho2018	|	0	(omitted)				
Illinois2009	|	-5.666955	3.784161	-1.50	0.135	-13.10004	1.766133
Illinois2010	|	-5.910597	4.25884	-1.39	0.166	-14.27608	2.454884
Illinois2011	|	-6.725834	4.115933	-1.63	0.103	-14.81061	1.358942
Illinois2012	|	-4.01237	3.448031	-1.16	0.245	-10.78521	2.760469
Illinois2013	|	-8.160763	2.904391	-2.81	0.005	-13.86575	-2.455775
Illinois2014	|	-6.520922	3.253611	-2.00	0.046	-12.91187	-.1299736
Illinois2015	|	-3.453535	2.905364	-1.19	0.235	-9.160433	2.253364
Illinois2016	|	-5.439685	1.736147	-3.13	0.002	-8.849933	-2.029436
Illinois2017	|	-4.725758	2.49119	-1.90	0.058	-9.619111	.1675936
Illinois2018	|	0	(omitted)				
Indiana2009	|	5.986633	6.747616	0.89	0.375	-7.26746	19.24073
Indiana2010	|	5.000472	6.499953	0.77	0.442	-7.767145	17.76809
Indiana2011	|	7.253224	7.788073	0.93	0.352	-8.044601	22.55105
Indiana2012	|	2.560793	6.735932	0.38	0.704	-10.67035	15.79194
Indiana2013	|	-.2817	5.78214	-0.05	0.961	-11.63934	11.07594
Indiana2014	|	-.1645594	6.468376	-0.03	0.980	-12.87015	12.54103
Indiana2015	|	.0629697	4.912814	0.01	0.990	-9.58709	9.71303
Indiana2016	|	-.0372328	6.12305	-0.01	0.995	-12.06451	11.99005
Indiana2017	|	4.710643	7.707984	0.61	0.541	-10.42987	19.85115
Indiana2018	|	0	(omitted)				
Iowa2009	|	8.298109	2.765663	3.00	0.003	2.86562	13.7306
Iowa2010	|	2.971048	2.919698	1.02	0.309	-2.764007	8.706102
Iowa2011	|	7.12847	2.699102	2.64	0.008	1.826724	12.43022
Iowa2012	|	3.706549	3.158406	1.17	0.241	-2.497392	9.910489
Iowa2013	|	4.337173	3.312837	1.31	0.191	-2.170111	10.84446
Iowa2014	|	.2999373	3.35924	0.09	0.929	-6.298494	6.898369
Iowa2015	|	2.793598	2.475406	1.13	0.260	-2.06875	7.655946
Iowa2016	|	-1.247709	.8846551	-1.41	0.159	-2.985405	.4899858
Iowa2017	|	-3.031635	2.655484	-1.14	0.254	-8.247704	2.184434
Iowa2018	|	0	(omitted)				
Kansas2009	|	7.834673	2.43361	3.22	0.001	3.054422	12.61492
Kansas2010	|	3.112604	2.935621	1.06	0.289	-2.653729	8.878937
Kansas2011	|	4.921797	2.607526	1.89	0.060	-.2000696	10.04366
Kansas2012	|	5.247529	2.533245	2.07	0.039	.271569	10.22349
Kansas2013	|	2.475547	2.260265	1.10	0.274	-1.964208	6.915302
Kansas2014	|	1.433474	2.733958	0.52	0.600	-3.936739	6.803686
Kansas2015	|	1.819909	2.303625	0.79	0.430	-2.705017	6.344835
Kansas2016	|	-.5441277	.9312901	-0.58	0.559	-2.373426	1.285171
Kansas2017	|	-4.789659	2.009958	-2.38	0.018	-8.737745	-.8415723
Kansas2018	|	0	(omitted)				
Kentucky2009	|	-18.88052	4.003388	-4.72	0.000	-26.74422	-11.01681
Kentucky2010	|	-15.93357	4.018332	-3.97	0.000	-23.82663	-8.040512
Kentucky2011	|	-17.3422	4.409517	-3.93	0.000	-26.00365	-8.680746
Kentucky2012	|	-9.441922	4.402716	-2.14	0.032	-18.09001	-.7938288
Kentucky2013	|	-16.21529	5.009575	-3.24	0.001	-26.05542	-6.375171
Kentucky2014	|	-13.22562	3.890321	-3.40	0.001	-20.86724	-5.58401

Kentucky2015	-5.286665	3.751622	-1.41	0.159	-12.65584	2.082508
Kentucky2016	-5.648077	3.066001	-1.84	0.066	-11.67051	.3743556
Kentucky2017	.2381202	3.496352	0.07	0.946	-6.629634	7.105875
Kentucky2018	0	(omitted)				
Louisiana2009	-8.11577	3.038043	-2.67	0.008	-14.08329	-2.148255
Louisiana2010	-5.10155	3.654029	-1.40	0.163	-12.27902	2.075924
Louisiana2011	-7.612088	5.507517	-1.38	0.167	-18.4303	3.206125
Louisiana2012	-6.395085	3.255445	-1.96	0.050	-12.78964	-.0005348
Louisiana2013	-1.128367	4.104418	-0.27	0.783	-9.190524	6.93379
Louisiana2014	-4.148021	4.07616	-1.02	0.309	-12.15467	3.85863
Louisiana2015	-2.747743	2.693013	-1.02	0.308	-8.037529	2.542044
Louisiana2016	-4.886611	2.427131	-2.01	0.045	-9.654134	-.1190878
Louisiana2017	-4.333181	2.550959	-1.70	0.090	-9.343936	.6775739
Louisiana2018	0	(omitted)				
Maine2009	-8.19772	3.520399	-2.33	0.020	-15.11271	-1.282729
Maine2012	-6.603349	2.847622	-2.32	0.021	-12.19683	-1.009871
Maine2013	-12.44045	4.16671	-2.99	0.003	-20.62497	-4.255936
Maine2014	-7.380499	3.494529	-2.11	0.035	-14.24467	-.5163255
Maine2015	-1.644197	3.31703	-0.50	0.620	-8.159716	4.871322
Maine2016	-1.771291	1.494307	-1.19	0.236	-4.706503	1.163921
Maine2017	7.15266	2.176161	3.29	0.001	2.878107	11.42721
Maine2018	0	(omitted)				
Maryland2009	-20.4847	6.065635	-3.38	0.001	-32.39921	-8.570201
Maryland2010	-22.51003	7.102753	-3.17	0.002	-36.4617	-8.558352
Maryland2011	-21.63149	6.837491	-3.16	0.002	-35.06212	-8.200861
Maryland2012	-21.17447	6.08525	-3.48	0.001	-33.1275	-9.221436
Maryland2013	-21.18236	5.988411	-3.54	0.000	-32.94518	-9.419549
Maryland2014	-18.84313	5.553	-3.39	0.001	-29.75069	-7.935582
Maryland2015	-13.94955	4.853113	-2.87	0.004	-23.48234	-4.416754
Maryland2016	-6.532757	5.252546	-1.24	0.214	-16.85014	3.784626
Maryland2017	-4.636301	3.144945	-1.47	0.141	-10.8138	1.541198
Maryland2018	0	(omitted)				
Massachusetts2009	-16.8075	3.42	-4.91	0.000	-23.52528	-10.08972
Massachusetts2010	-19.06344	3.387716	-5.63	0.000	-25.71781	-12.40908
Massachusetts2011	-19.85712	4.006464	-4.96	0.000	-27.72687	-11.98737
Massachusetts2012	-17.45998	3.196747	-5.46	0.000	-23.73923	-11.18073
Massachusetts2013	-15.28883	2.918145	-5.24	0.000	-21.02084	-9.556827
Massachusetts2014	-13.2737	3.397496	-3.91	0.000	-19.94727	-6.600122
Massachusetts2015	-4.359247	2.92346	-1.49	0.136	-10.10169	1.383197
Massachusetts2016	-.8239097	2.308244	-0.36	0.721	-5.357909	3.71009
Massachusetts2017	-6.425717	3.179155	-2.02	0.044	-12.67041	-.1810196
Massachusetts2018	0	(omitted)				
Michigan2009	-2.424729	3.65532	-0.66	0.507	-9.604739	4.755281
Michigan2010	-3.720966	4.074241	-0.91	0.361	-11.72385	4.281915
Michigan2011	-3.635499	3.732554	-0.97	0.330	-10.96722	3.696219
Michigan2012	-5.050744	3.743427	-1.35	0.178	-12.40382	2.302331
Michigan2013	-5.257779	3.503927	-1.50	0.134	-12.14041	1.624856
Michigan2014	-6.329717	4.05504	-1.56	0.119	-14.29488	1.635449
Michigan2015	-2.317171	3.276822	-0.71	0.480	-8.753711	4.119369
Michigan2016	-.9150924	1.658592	-0.55	0.581	-4.173004	2.342819
Michigan2017	-2.024161	2.268572	-0.89	0.373	-6.480234	2.431912
Michigan2018	0	(omitted)				
Minnesota2009	9.470558	3.549719	2.67	0.008	2.497976	16.44314
Minnesota2010	7.595525	3.875684	1.96	0.051	-.0173382	15.20839
Minnesota2011	7.532996	3.820019	1.97	0.049	.0294731	15.03652
Minnesota2012	7.563859	3.521553	2.15	0.032	.6466023	14.48112
Minnesota2013	5.186198	3.554466	1.46	0.145	-1.795709	12.16811
Minnesota2014	.1093295	3.888199	0.03	0.978	-7.528116	7.746775
Minnesota2015	6.352482	3.26464	1.95	0.052	-.060129	12.76509
Minnesota2016	3.06891	1.910095	1.61	0.109	-.6830185	6.820838
Minnesota2017	.8960649	2.733686	0.33	0.743	-4.473613	6.265743
Minnesota2018	0	(omitted)				
Mississippi2015	4.245548	2.779691	1.53	0.127	-1.214495	9.705592
Mississippi2016	-.6622336	1.655903	-0.40	0.689	-3.914863	2.590396
Mississippi2017	-.3479345	2.300958	-0.15	0.880	-4.867621	4.171752
Mississippi2018	0	(omitted)				
Missouri2009	-10.36556	7.298118	-1.42	0.156	-24.70099	3.96986
Missouri2010	-9.542442	7.710243	-1.24	0.216	-24.68739	5.602503
Missouri2011	-9.945848	6.087986	-1.63	0.103	-21.90425	2.012558
Missouri2012	-13.43681	6.818091	-1.97	0.049	-26.82933	-.0442822
Missouri2013	-13.04025	5.654083	-2.31	0.021	-24.14635	-1.934141
Missouri2014	-12.29101	5.702345	-2.16	0.032	-23.49192	-1.090106
Missouri2015	-10.46085	5.830908	-1.79	0.073	-21.91429	.9925851
Missouri2016	-6.220676	2.55782	-2.43	0.015	-11.24491	-1.196445
Missouri2017	-9.414358	2.681378	-3.51	0.000	-14.68129	-4.147425
Missouri2018	0	(omitted)				

Nebraska2009	12.27961	2.719868	4.51	0.000	6.937077	17.62215
Nebraska2010	8.634653	2.95572	2.92	0.004	2.828842	14.44046
Nebraska2011	7.483511	2.681465	2.79	0.005	2.216409	12.75061
Nebraska2012	7.856626	2.563908	3.06	0.002	2.820436	12.89282
Nebraska2014	1.705934	2.707602	0.63	0.529	-3.612509	7.024377
Nebraska2015	3.118696	2.304935	1.35	0.177	-1.408803	7.646195
Nebraska2016	-2.04473	.8562533	-2.39	0.017	-3.726637	-.3628237
Nebraska2017	-4.155944	1.930079	-2.15	0.032	-7.947127	-.3647614
Nebraska2018	0	(omitted)				
Nevada2009	11.29474	3.428956	3.29	0.001	4.559372	18.03012
Nevada2010	3.914383	8.76587	0.45	0.655	-13.30409	21.13286
Nevada2011	12.12512	3.494239	3.47	0.001	5.261511	18.98872
Nevada2012	10.41961	2.993239	3.48	0.001	4.540104	16.29912
Nevada2013	6.648759	2.784501	2.39	0.017	1.179267	12.11825
Nevada2014	-2.588231	5.219171	-0.50	0.620	-12.84005	7.663593
Nevada2015	6.055987	2.488647	2.43	0.015	1.16763	10.94434
Nevada2016	2.644706	1.456448	1.82	0.070	-.2161413	5.505554
Nevada2017	-.6199594	2.290874	-0.27	0.787	-5.119839	3.87992
Nevada2018	0	(omitted)				
New Hampshire2009	-18.84765	3.773973	-4.99	0.000	-26.26073	-11.43458
New Hampshire2010	-21.92696	3.212352	-6.83	0.000	-28.23686	-15.61705
New Hampshire2011	-17.35461	3.379065	-5.14	0.000	-23.99199	-10.71724
New Hampshire2012	-19.50337	3.456772	-5.64	0.000	-26.29338	-12.71336
New Hampshire2013	-19.709	3.82702	-5.15	0.000	-27.22628	-12.19173
New Hampshire2014	-6.275191	4.474601	-1.40	0.161	-15.06449	2.514103
New Hampshire2015	.7915658	3.024424	0.26	0.794	-5.149199	6.732331
New Hampshire2016	1.111197	3.086244	0.36	0.719	-4.950999	7.173393
New Hampshire2017	-5.56968	3.179898	-1.75	0.080	-11.81584	.676476
New Hampshire2018	0	(omitted)				
New Jersey2010	-19.69748	4.251048	-4.63	0.000	-28.04766	-11.3473
New Jersey2011	-18.97947	3.995283	-4.75	0.000	-26.82725	-11.13168
New Jersey2012	-16.90221	3.914192	-4.32	0.000	-24.59071	-9.213704
New Jersey2013	-18.23628	3.387734	-5.38	0.000	-24.89068	-11.58188
New Jersey2014	-19.69381	3.9648	-4.97	0.000	-27.48172	-11.9059
New Jersey2015	-16.40421	3.270675	-5.02	0.000	-22.82867	-9.979739
New Jersey2016	-13.93055	2.908274	-4.79	0.000	-19.64317	-8.217939
New Jersey2017	-8.307132	2.364101	-3.51	0.000	-12.95085	-3.663415
New Jersey2018	0	(omitted)				
New Mexico2009	2.85407	2.703239	1.06	0.292	-2.455802	8.163941
New Mexico2010	.5350722	3.036115	0.18	0.860	-5.428657	6.498801
New Mexico2011	1.59234	2.770595	0.57	0.566	-3.849837	7.034517
New Mexico2012	8.167047	2.799265	2.92	0.004	2.668555	13.66554
New Mexico2013	2.322616	3.35214	0.69	0.489	-4.261868	8.9071
New Mexico2014	11.43176	5.209602	2.19	0.029	1.198729	21.66478
New Mexico2015	6.042322	3.598378	1.68	0.094	-1.02584	13.11048
New Mexico2016	1.63389	1.100057	1.49	0.138	-.526912	3.794692
New Mexico2017	-3.006227	2.434974	-1.23	0.218	-7.789157	1.776703
New Mexico2018	0	(omitted)				
New York2009	-3.661703	3.31458	-1.10	0.270	-10.17241	2.849004
New York2010	-5.118284	3.194031	-1.60	0.110	-11.3922	1.155632
New York2011	-3.464951	2.987433	-1.16	0.247	-9.333056	2.403154
New York2012	-2.589753	2.862182	-0.90	0.366	-8.211832	3.032326
New York2013	-4.546237	2.627878	-1.73	0.084	-9.70808	.6156063
New York2014	-5.8981	3.096895	-1.90	0.057	-11.98122	.1850159
New York2015	-1.15374	2.760902	-0.42	0.676	-6.576877	4.269397
New York2016	.0527525	1.769825	0.03	0.976	-3.42365	3.529155
New York2017	-2.595689	2.289804	-1.13	0.257	-7.093467	1.902088
New York2018	0	(omitted)				
North Carolina2009	-2.053124	3.214105	-0.64	0.523	-8.366472	4.260225
North Carolina2010	-2.793357	3.318058	-0.84	0.400	-9.310896	3.724183
North Carolina2011	-2.917485	2.978599	-0.98	0.328	-8.768237	2.933266
North Carolina2012	-3.554315	3.012703	-1.18	0.239	-9.472056	2.363425
North Carolina2013	-5.232427	2.836875	-1.84	0.066	-10.8048	.3399422
North Carolina2014	-5.69456	3.171793	-1.80	0.073	-11.9248	.5356757
North Carolina2015	-1.82119	2.680088	-0.68	0.497	-7.085589	3.443208
North Carolina2016	-3.844191	1.818548	-2.11	0.035	-7.416298	-.2720845
North Carolina2017	.4225719	2.349395	0.18	0.857	-4.192257	5.037401
North Carolina2018	0	(omitted)				
North Dakota2016	0	(omitted)				
Ohio2009	-18.61333	3.822429	-4.87	0.000	-26.12159	-11.10508
Ohio2010	-14.60943	4.06877	-3.59	0.000	-22.60156	-6.61729
Ohio2011	-14.68531	3.664303	-4.01	0.000	-21.88296	-7.487655
Ohio2012	-12.52974	3.722994	-3.37	0.001	-19.84268	-5.216803
Ohio2013	-13.97473	3.357824	-4.16	0.000	-20.57038	-7.37908
Ohio2014	-7.885877	3.333762	-2.37	0.018	-14.43426	-1.337492
Ohio2015	1.495753	2.912018	0.51	0.608	-4.224217	7.215722

Ohio2016	4.640457	2.285135	2.03	0.043	.1518499	9.129064
Ohio2017	6.136081	2.855123	2.15	0.032	.5278683	11.74429
Ohio2018	0	(omitted)				
Oklahoma2009	15.33862	3.151345	4.87	0.000	9.148549	21.52869
Oklahoma2010	14.32159	3.221574	4.45	0.000	7.993574	20.64961
Oklahoma2011	9.541663	3.168847	3.01	0.003	3.317214	15.76611
Oklahoma2012	11.78723	2.92234	4.03	0.000	6.046988	17.52748
Oklahoma2013	8.518374	2.539947	3.35	0.001	3.529249	13.5075
Oklahoma2014	-2.832553	4.179748	-0.68	0.498	-11.04268	5.377572
Oklahoma2015	6.226535	2.402828	2.59	0.010	1.50675	10.94632
Oklahoma2016	3.356174	.9603273	3.49	0.001	1.469839	5.242509
Oklahoma2017	-1.037615	2.022126	-0.51	0.608	-5.009602	2.934373
Oklahoma2018	0	(omitted)				
Oregon2009	11.9347	2.901636	4.11	0.000	6.235121	17.63427
Oregon2010	7.591683	3.111499	2.44	0.015	1.47988	13.70349
Oregon2011	9.352334	2.944037	3.18	0.002	3.569471	15.1352
Oregon2012	9.015305	3.143947	2.87	0.004	2.839766	15.19084
Oregon2013	4.339609	2.982858	1.45	0.146	-1.519509	10.19873
Oregon2014	2.354459	3.092725	0.76	0.447	-3.720467	8.429384
Oregon2015	3.528024	2.685262	1.31	0.189	-1.746536	8.802585
Oregon2016	-2.632707	2.473381	-1.06	0.288	-7.491077	2.225664
Oregon2017	-3.292847	2.067778	-1.59	0.112	-7.354508	.768814
Oregon2018	0	(omitted)				
Pennsylvania2009	-8.060292	3.638816	-2.22	0.027	-15.20788	-.9127003
Pennsylvania2010	-10.81304	4.043896	-2.67	0.008	-18.75632	-2.869768
Pennsylvania2011	-11.16763	3.094935	-3.61	0.000	-17.24689	-5.08836
Pennsylvania2012	-12.42032	3.150963	-3.94	0.000	-18.60964	-6.230998
Pennsylvania2013	-11.03759	3.121656	-3.54	0.000	-17.16935	-4.905839
Pennsylvania2014	-12.75425	3.595481	-3.55	0.000	-19.81672	-5.691776
Pennsylvania2015	-6.715143	3.283486	-2.05	0.041	-13.16477	-.2655128
Pennsylvania2016	-.1260746	3.347391	-0.04	0.970	-6.70123	6.449081
Pennsylvania2017	-3.447446	3.6969	-0.93	0.351	-10.70913	3.814239
Pennsylvania2018	0	(omitted)				
Rhode Island2009	-8.470549	2.458987	-3.44	0.001	-13.30065	-3.640452
Rhode Island2010	-9.887093	3.199457	-3.09	0.002	-16.17167	-3.602517
Rhode Island2011	-8.88462	3.007917	-2.95	0.003	-14.79296	-2.97628
Rhode Island2012	-6.571147	2.771927	-2.37	0.018	-12.01594	-1.126353
Rhode Island2013	-4.371268	2.391343	-1.83	0.068	-9.068495	.3259593
Rhode Island2014	-5.745612	4.505313	-1.28	0.203	-14.59523	3.104007
Rhode Island2015	.6200534	3.348019	0.19	0.853	-5.956337	7.196444
Rhode Island2016	-3.648819	2.714649	-1.34	0.179	-8.981104	1.683466
Rhode Island2017	-6.931428	3.515619	-1.97	0.049	-13.83703	-.0258283
Rhode Island2018	0	(omitted)				
South Carolina2009	2.851309	4.118548	0.69	0.489	-5.238602	10.94122
South Carolina2010	.9725135	4.556392	0.21	0.831	-7.977439	9.922466
South Carolina2011	1.476197	3.765451	0.39	0.695	-5.920139	8.872532
South Carolina2012	.0630432	2.962928	0.02	0.983	-5.756927	5.883013
South Carolina2013	-4.157869	2.81359	-1.48	0.140	-9.6845	1.368762
South Carolina2014	-3.336485	3.169028	-1.05	0.293	-9.56129	2.888319
South Carolina2015	-1.013623	2.917161	-0.35	0.728	-6.743694	4.716448
South Carolina2016	-3.010843	1.887121	-1.60	0.111	-6.717644	.6959592
South Carolina2017	-4.890367	2.363733	-2.07	0.039	-9.53336	-.2473734
South Carolina2018	0	(omitted)				
Tennessee2009	-7.138902	4.624611	-1.54	0.123	-16.22285	1.945051
Tennessee2010	-6.819442	4.722289	-1.44	0.149	-16.09526	2.456376
Tennessee2011	-8.058708	5.054035	-1.59	0.111	-17.98616	1.868747
Tennessee2012	-6.134877	4.95289	-1.24	0.216	-15.86366	3.593902
Tennessee2013	-9.482574	6.088331	-1.56	0.120	-21.44166	2.476509
Tennessee2014	-8.719197	4.001289	-2.18	0.030	-16.57878	-.8596117
Tennessee2015	-3.928608	3.21559	-1.22	0.222	-10.24487	2.387657
Tennessee2016	-4.348944	2.038029	-2.13	0.033	-8.352168	-.34572
Tennessee2017	-6.476192	2.720467	-2.38	0.018	-11.8199	-1.13248
Tennessee2018	0	(omitted)				
Texas2009	5.094937	3.025664	1.68	0.093	-.8482619	11.03814
Texas2010	2.759318	3.654713	0.76	0.451	-4.4195	9.938136
Texas2011	2.534602	3.231655	0.78	0.433	-3.813219	8.882423
Texas2012	3.253071	2.960421	1.10	0.272	-2.561976	9.068117
Texas2013	-.2754272	2.646976	-0.10	0.917	-5.474785	4.92393
Texas2014	-.5724811	2.953909	-0.19	0.846	-6.374734	5.229772
Texas2015	1.975323	2.391992	0.83	0.409	-2.723178	6.673824
Texas2016	-1.193779	.8787202	-1.36	0.175	-2.919817	.5322582
Texas2017	-3.756959	1.997195	-1.88	0.060	-7.679975	.1660576
Texas2018	0	(omitted)				
Utah2009	7.505055	2.813224	2.67	0.008	1.979143	13.03097
Utah2010	3.247107	3.35659	0.97	0.334	-3.346119	9.840332
Utah2011	5.312508	3.395609	1.56	0.118	-1.357362	11.98238

Utah2012	6.9243	2.796585	2.48	0.014	1.43107	12.41753
Utah2013	1.747684	2.544276	0.69	0.492	-3.249943	6.745312
Utah2014	3.751642	2.923723	1.28	0.200	-1.99132	9.494604
Utah2015	5.289472	2.641537	2.00	0.046	.1007982	10.47815
Utah2016	.1291874	1.19539	0.11	0.914	-2.218872	2.477247
Utah2017	-6.926834	3.627037	-1.91	0.057	-14.05129	.1976207
Utah2018	0	(omitted)				
Vermont2015	20.1952	1.420768	14.21	0.000	17.40444	22.98596
Vermont2016	19.84232	1.534154	12.93	0.000	16.82883	22.8558
Vermont2017	0	(omitted)				
Vermont2018	0	(omitted)				
Virginia2009	-.6785798	3.367103	-0.20	0.840	-7.292456	5.935296
Virginia2010	-4.324672	3.927765	-1.10	0.271	-12.03984	3.390492
Virginia2011	-2.333876	3.704631	-0.63	0.529	-9.610746	4.942994
Virginia2012	-2.165713	3.278467	-0.66	0.509	-8.605486	4.274059
Virginia2013	-4.080501	3.158736	-1.29	0.197	-10.28509	2.124087
Virginia2014	-4.196855	4.002108	-1.05	0.295	-12.05805	3.664337
Virginia2015	-2.303032	3.931074	-0.59	0.558	-10.02469	5.418631
Virginia2016	.9102905	2.173957	0.42	0.676	-3.359934	5.180515
Virginia2017	-3.276155	3.180639	-1.03	0.303	-9.523768	2.971457
Virginia2018	0	(omitted)				
Washington2009	8.901994	2.915287	3.05	0.002	3.175604	14.62838
Washington2010	5.516879	3.344304	1.65	0.100	-1.052214	12.08597
Washington2011	6.895851	2.763184	2.50	0.013	1.46823	12.32347
Washington2012	3.876523	3.803648	1.02	0.309	-3.594842	11.34789
Washington2013	2.127624	2.378077	0.89	0.371	-2.543544	6.798791
Washington2014	1.205295	2.870851	0.42	0.675	-4.433811	6.844401
Washington2015	3.029216	2.646235	1.14	0.253	-2.168686	8.227118
Washington2016	-1.005253	1.57893	-0.64	0.525	-4.106687	2.096181
Washington2017	-4.837511	2.108477	-2.29	0.022	-8.979115	-.695908
Washington2018	0	(omitted)				
West Virginia2010	-17.3665	15.29143	-1.14	0.257	-47.40289	12.6699
West Virginia2011	-5.1599	15.48195	-0.33	0.739	-35.57053	25.25073
West Virginia2012	-18.72737	16.10833	-1.16	0.245	-50.36837	12.91363
West Virginia2013	-18.57919	10.14689	-1.83	0.068	-38.51036	1.351976
West Virginia2014	-12.77243	11.56267	-1.10	0.270	-35.48455	9.939686
West Virginia2015	-4.195598	9.13018	-0.46	0.646	-22.12967	13.73848
West Virginia2016	5.467663	6.918483	0.79	0.430	-8.122057	19.05738
West Virginia2017	13.812	8.063734	1.71	0.087	-2.02729	29.6513
West Virginia2018	0	(omitted)				
Wisconsin2009	0	(omitted)				
Wisconsin2010	0	(omitted)				
Wisconsin2011	0	(omitted)				
Wisconsin2012	0	(omitted)				
Wisconsin2013	0	(omitted)				
Wisconsin2014	0	(omitted)				
Wisconsin2015	0	(omitted)				
Wisconsin2016	0	(omitted)				
Wisconsin2017	0	(omitted)				
Wisconsin2018	0	(omitted)				
_cons	136.3169	91.87683	1.48	0.138	-44.15336	316.7871
sigma_u	19.685092					
sigma_e	5.7871972					
rho	.92044635	(fraction of variance due to u_i)				

COUNTY AND YEAR FIXED EFFECTS FOR CVD DEATH RATE (TABLE 2 SPECIFICATION 1)

Fixed-effects (within) regression		Number of obs	= 31,012
Group variable: county_fip~e		Number of groups	= 3,150
R-sq:		Obs per group:	
within = 0.1569		min	= 1
between = 0.0012		avg	= 9.8
overall = 0.0159		max	= 10
		F(28,3149)	= 89.36
corr(u_i, Xb) = -0.1280		Prob > F	= 0.0000

(Std. Err. adjusted for 3,150 clusters in county_fipscode)

cdc_heart_disease_~h	Coef.	Robust Std. Err.	t	P>|t|	[95% Conf.	Interval]
acs_pct_female	.3409742	.4329923	0.79	0.431	-.5080014	1.18995
acs_median_age	1.536069	.3724445	4.12	0.000	.8058101	2.266327
acs_pct_black	-.0107354	.2413531	-0.04	0.965	-.4839606	.4624899
acs_pct_white	-.0717861	.1247179	-0.58	0.565	-.3163227	.1727505
acs_pct_hispan	.2419456	.4314109	0.56	0.575	-.6039294	1.087821
acs_pct_asian	-.1054069	.7548355	-0.14	0.889	-1.585426	1.374612
acs_pct_bachelor_dgr	-.3156336	.2645122	-1.19	0.233	-.8342674	.2030002
acs_median_hh_income	-.0000478	.000145	-0.33	0.742	-.0003321	.0002366
acs_pct_person_inc99	-.1662807	.1749954	-0.95	0.342	-.5093973	.1768359
acs_pct_unemploy	-.0497029	.2940154	-0.17	0.866	-.6261841	.5267782
acs_pct_grp_qrt	.339881	.2730854	1.24	0.213	-.1955622	.8753243
acs_pct_va	-.4632768	.3455583	-1.34	0.180	-1.140819	.2142655
acs_pct_armed_forces	1.62571	.4766393	3.41	0.001	.6911547	2.560265
ahrf_fed_hlth_cnt	-.1742908	.0752576	-2.32	0.021	-.3218498	-.0267318
ahrf_rural_h_clinic	-.4186592	.4115517	-1.02	0.309	-1.225596	.3882775
acs_total_household	.0000378	.0000815	0.46	0.643	-.000122	.0001976
acs_pct_rented_hh	.1158973	.1644035	0.70	0.481	-.2064516	.4382461
acs_pct_mobile_home	-.0127164	.2073848	-0.06	0.951	-.4193395	.3939067
acs_total_pop_wt	-.0000121	.0000238	-0.51	0.610	-.0000587	.0000345
year						
2010	-5.879618	.9785821	-6.01	0.000	-7.798341	-3.960894
2011	-13.07198	.8289839	-15.77	0.000	-14.69738	-11.44657
2012	-17.47782	1.098018	-15.92	0.000	-19.63072	-15.32491
2013	-20.86791	1.30212	-16.03	0.000	-23.421	-18.31482
2014	-22.49694	1.487577	-15.12	0.000	-25.41365	-19.58022
2015	-22.61685	1.656489	-13.65	0.000	-25.86476	-19.36895
2016	-23.29069	1.894173	-12.30	0.000	-27.00462	-19.57675
2017	-23.56686	2.143632	-10.99	0.000	-27.76992	-19.3638
2018	-25.53	2.397627	-10.65	0.000	-30.23107	-20.82893
_cons	326.9273	30.95671	10.56	0.000	266.2299	387.6247
sigma_u	56.78993					
sigma_e	22.02403					
rho	.86926206	(fraction of variance due to u_i)				

COUNTY, YEAR, & STATE-BY-YEAR FIXED EFFECTS FOR CVD DEATH RATE (TABLE 2 SPECIFICATION 2)

```
Fixed-effects (within) regression               Number of obs     =     31,012
Group variable: county_fip~e                    Number of groups  =      3,150

R-sq:                                           Obs per group:
     within  = 0.1814                                         min =          1
     between = 0.1238                                         avg =        9.8
     overall = 0.1413                                         max =         10

                                                F(458,3149)       =    4741.46
corr(u_i, Xb)  = 0.1159                         Prob > F          =     0.0000

                              (Std. Err. adjusted for 3,150 clusters in county_fipscode)
```

cdc_heart_disease_de~h	Coef.	Robust Std. Err.	t	P>|t|	[95% Conf.	Interval]
acs_pct_female	.3979829	.4319126	0.92	0.357	-.4488757	1.244842
acs_median_age	.9442199	.4001888	2.36	0.018	.1595627	1.728877
acs_pct_black	.1794991	.2397232	0.75	0.454	-.2905303	.6495285
acs_pct_white	-.2094997	.1254058	-1.67	0.095	-.455385	.0363857
acs_pct_hispan	.0770919	.461046	0.17	0.867	-.8268891	.981073
acs_pct_asian	.3603354	.7752729	0.46	0.642	-1.159756	1.880427
acs_pct_bachelor_dgr	-.2792628	.263056	-1.06	0.288	-.7950413	.2365157
acs_median_hh_income	-.0002255	.0001522	-1.48	0.138	-.0005239	.0000728
acs_pct_person_inc99	-.1712101	.1814317	-0.94	0.345	-.5269464	.1845261
acs_pct_unemploy	.0870857	.3142549	0.28	0.782	-.5290795	.7032508
acs_pct_grp_qrt	.3501566	.2665062	1.31	0.189	-.1723869	.8727
acs_pct_va	-.1184386	.3484473	-0.34	0.734	-.8016453	.5647681
acs_pct_armed_forces	1.298195	.4737176	2.74	0.006	.3693691	2.227022
ahrf_fed_hlth_cnt	-.023582	.0711803	-0.33	0.740	-.1631464	.1159824
ahrf_rural_h_clinic	-.5452554	.4158551	-1.31	0.190	-1.36063	.2701191
acs_total_household	-.0000278	.0000833	-0.33	0.739	-.000191	.0001355
acs_pct_rented_hh	.15963	.1651907	0.97	0.334	-.1642622	.4835222
acs_pct_mobile_home	.0083726	.2116054	0.04	0.968	-.4065259	.4232711
acs_total_pop_wt	-9.09e-06	.0000239	-0.38	0.704	-.000056	.0000378
year						
2010	-5.707472	4.53021	-1.26	0.208	-14.58993	3.17499
2011	-17.18498	6.121473	-2.81	0.005	-29.18746	-5.182496
2012	-17.32925	6.911407	-2.51	0.012	-30.88057	-3.777932
2013	-21.79299	8.377083	-2.60	0.009	-38.21808	-5.36789
2014	-17.28065	7.452705	-2.32	0.020	-31.8933	-2.668003
2015	-21.26258	8.484871	-2.51	0.012	-37.89901	-4.626141
2016	-17.91365	7.973233	-2.25	0.025	-33.54691	-2.280389
2017	-21.76537	9.874971	-2.20	0.028	-41.1274	-2.403342
2018	-22.51939	8.685079	-2.59	0.010	-39.54838	-5.490401
state_year_num						
Alabama2010	2.072172	4.990205	0.42	0.678	-7.71221	11.85655
Alabama2011	9.697211	6.863953	1.41	0.158	-3.761062	23.15548
Alabama2012	2.219945	7.731686	0.29	0.774	-12.93971	17.3796
Alabama2013	1.596966	9.217041	0.17	0.862	-16.47505	19.66898
Alabama2014	-7.766198	8.543658	-0.91	0.363	-24.5179	8.985504
Alabama2015	.1053774	9.435286	0.01	0.991	-18.39455	18.60531
Alabama2016	-5.498019	8.887112	-0.62	0.536	-22.92314	11.9271
Alabama2017	-1.548682	10.71445	-0.14	0.885	-22.55668	19.45932
Alabama2018	-4.95405	9.686022	-0.51	0.609	-23.9456	14.0375
Alaska2009	1.061824	14.52395	0.07	0.942	-27.41553	29.53918
Alaska2010	19.34471	14.55774	1.33	0.184	-9.198904	47.88833
Alaska2011	24.24133	13.62243	1.78	0.075	-2.468416	50.95107
Alaska2012	10.04568	14.54087	0.69	0.490	-18.46486	38.55623
Alaska2013	1.911391	12.02788	0.16	0.874	-21.67189	25.49467
Alaska2014	-10.75251	11.26952	-0.95	0.340	-32.84885	11.34384
Alaska2015	-.9997054	9.940001	-0.10	0.920	-20.48924	18.48983
Alaska2016	2.219856	8.258867	0.27	0.788	-13.97345	18.41316
Alaska2017	8.420414	6.54201	1.29	0.198	-4.406619	21.24745
Alaska2018	0	(omitted)				
Arizona2009	-9.300394	9.371187	-0.99	0.321	-27.67465	9.073858
Arizona2010	-15.75053	9.700822	-1.62	0.105	-34.7711	3.270044
Arizona2011	-3.303889	7.887166	-0.42	0.675	-18.7684	12.16062
Arizona2012	-4.477725	7.204676	-0.62	0.534	-18.60406	9.64861
Arizona2013	-.9242952	6.575355	-0.14	0.888	-13.81671	11.96812

Arizona2014	-9.186741	5.924683	-1.55	0.121	-20.80337	2.42989
Arizona2015	-6.450192	5.518776	-1.17	0.243	-17.27095	4.37057
Arizona2016	-6.99532	5.612069	-1.25	0.213	-17.999	4.008363
Arizona2017	-1.654316	4.3607	-0.38	0.704	-10.20442	6.895787
Arizona2018	0	(omitted)				
Arkansas2009	.745734	10.2854	0.07	0.942	-19.42102	20.91249
Arkansas2010	1.953096	9.604929	0.20	0.839	-16.87946	20.78565
Arkansas2011	3.870332	8.767734	0.44	0.659	-13.32072	21.06138
Arkansas2012	-1.226631	7.793855	-0.16	0.875	-16.50818	14.05492
Arkansas2013	-.8217233	7.213693	-0.11	0.909	-14.96574	13.32229
Arkansas2014	-6.731029	6.518154	-1.03	0.302	-19.51129	6.04923
Arkansas2015	-1.274651	6.206051	-0.21	0.837	-13.44296	10.89366
Arkansas2016	-3.238254	6.247244	-0.52	0.604	-15.48734	9.010827
Arkansas2017	4.588617	4.039144	1.14	0.256	-3.331004	12.50824
Arkansas2018	0	(omitted)				
California2009	4.731449	8.957414	0.53	0.597	-12.83151	22.29441
California2010	3.010441	8.553665	0.35	0.725	-13.76088	19.78176
California2011	9.335211	7.871866	1.19	0.236	-6.099296	24.76972
California2012	6.521423	6.687415	0.98	0.330	-6.590709	19.63356
California2013	7.015431	6.015378	1.17	0.244	-4.779026	18.80989
California2014	-3.88494	5.576497	-0.70	0.486	-14.81888	7.048997
California2015	-.5974682	5.700789	-0.10	0.917	-11.77511	10.58017
California2016	-5.601411	5.741246	-0.98	0.329	-16.85837	5.655551
California2017	-.1039785	4.441356	-0.02	0.981	-8.812224	8.604266
California2018	0	(omitted)				
Colorado2009	5.900461	9.863958	0.60	0.550	-13.43998	25.2409
Colorado2010	4.080509	9.278341	0.44	0.660	-14.1117	22.27272
Colorado2011	11.19732	8.068447	1.39	0.165	-4.622625	27.01727
Colorado2012	6.066449	7.000593	0.87	0.386	-7.659738	19.79264
Colorado2013	5.891133	6.741837	0.87	0.382	-7.327706	19.10997
Colorado2014	-.8422072	7.108459	-0.12	0.906	-14.77989	13.09547
Colorado2015	2.766246	6.456716	0.43	0.668	-9.893551	15.42604
Colorado2016	.530805	6.505093	0.08	0.935	-12.22385	13.28546
Colorado2017	6.160405	4.354781	1.41	0.157	-2.37809	14.6989
Colorado2018	0	(omitted)				
Connecticut2009	7.462972	9.043052	0.83	0.409	-10.2679	25.19384
Connecticut2010	7.235042	8.48398	0.85	0.394	-9.399648	23.86973
Connecticut2011	15.07196	7.973677	1.89	0.059	-.5621698	30.70609
Connecticut2012	11.05003	6.661887	1.66	0.097	-2.01205	24.11211
Connecticut2013	12.41984	6.162945	2.02	0.044	.3360494	24.50364
Connecticut2014	2.470478	6.135096	0.40	0.687	-9.558713	14.49967
Connecticut2015	3.53465	5.392927	0.66	0.512	-7.039357	14.10866
Connecticut2016	-3.753438	5.606609	-0.67	0.503	-14.74642	7.239539
Connecticut2017	-.1630664	3.605292	-0.05	0.964	-7.232027	6.905894
Connecticut2018	0	(omitted)				
Delaware2009	3.716543	11.26351	0.33	0.741	-18.36802	25.80111
Delaware2010	-.1400663	9.78264	-0.01	0.989	-19.32106	19.04093
Delaware2011	9.206104	8.456709	1.09	0.276	-7.375115	25.78732
Delaware2012	1.77953	6.565331	0.27	0.786	-11.09323	14.65229
Delaware2013	.7313702	5.602355	0.13	0.896	-10.25327	11.71601
Delaware2014	-5.749548	4.850225	-1.19	0.236	-15.25947	3.760374
Delaware2015	-3.22531	5.326692	-0.61	0.545	-13.66945	7.21883
Delaware2016	-5.63654	5.879127	-0.96	0.338	-17.16385	5.890768
Delaware2017	-2.429172	4.164262	-0.58	0.560	-10.59411	5.735769
Delaware2018	0	(omitted)				
Dist. of Columbia2009	14.92386	8.991393	1.66	0.097	-2.705721	32.55344
Dist. of Columbia2010	5.379915	8.313358	0.65	0.518	-10.92023	21.68006
Dist. of Columbia2011	.3002757	7.369416	0.04	0.968	-14.14907	14.74962
Dist. of Columbia2012	-1.194254	6.089402	-0.20	0.845	-13.13385	10.74534
Dist. of Columbia2013	.7999609	5.441237	0.15	0.883	-9.868769	11.46869
Dist. of Columbia2014	.8535297	4.984089	0.17	0.864	-8.918862	10.62592
Dist. of Columbia2015	-.9834726	4.866771	-0.20	0.840	-10.52584	8.55889
Dist. of Columbia2016	-.5366227	5.396254	-0.10	0.921	-11.11715	10.04391
Dist. of Columbia2017	.5159655	3.481434	0.15	0.882	-6.310143	7.342074
Dist. of Columbia2018	0	(omitted)				
Florida2009	-4.349978	9.022869	-0.48	0.630	-22.04128	13.34132
Florida2010	-2.600832	8.54904	-0.30	0.761	-19.36308	14.16142
Florida2011	2.461241	7.414194	0.33	0.740	-12.0759	16.99838
Florida2012	-3.623616	6.344432	-0.57	0.568	-16.06326	8.816024
Florida2013	-5.533455	5.835347	-0.95	0.343	-16.97492	5.908013
Florida2014	-9.653955	5.342784	-1.81	0.071	-20.12965	.8217345
Florida2015	-4.924808	5.208322	-0.95	0.344	-15.13686	5.287242
Florida2016	-5.086636	5.584828	-0.91	0.362	-16.03691	5.863635
Florida2017	-1.500357	3.651295	-0.41	0.681	-8.659515	5.6588
Florida2018	0	(omitted)				
Georgia2009	12.16855	9.184186	1.32	0.185	-5.83905	30.17614

Georgia2010	8.595356	8.529907	1.01	0.314	-8.129383	25.32009
Georgia2011	10.0062	7.52908	1.33	0.184	-4.756197	24.7686
Georgia2012	-.515157	6.397286	-0.08	0.936	-13.05843	12.02811
Georgia2013	-.9935623	5.898729	-0.17	0.866	-12.5593	10.57218
Georgia2014	-6.256339	5.696766	-1.10	0.272	-17.42609	4.91341
Georgia2015	.0945493	5.754836	0.02	0.987	-11.18906	11.37816
Georgia2016	-2.851961	5.877185	-0.49	0.628	-14.37546	8.671539
Georgia2017	.4203092	3.872525	0.11	0.914	-7.172618	8.013237
Georgia2018	0	(omitted)				
Hawaii2009	9.731318	11.16657	0.87	0.384	-12.16316	31.6258
Hawaii2010	2.701116	9.914147	0.27	0.785	-16.73773	22.13996
Hawaii2011	7.336128	9.648003	0.76	0.447	-11.58088	26.25314
Hawaii2012	1.565291	7.892363	0.20	0.843	-13.9094	17.03999
Hawaii2013	10.88468	8.811225	1.24	0.217	-6.391646	28.161
Hawaii2014	4.8711	7.451482	0.65	0.513	-9.739152	19.48135
Hawaii2015	9.146609	6.414912	1.43	0.154	-3.431222	21.72444
Hawaii2016	-.9587962	5.533673	-0.17	0.862	-11.80877	9.891175
Hawaii2017	3.006836	4.364087	0.69	0.491	-5.549907	11.56358
Hawaii2018	0	(omitted)				
Idaho2009	-6.88738	10.26277	-0.67	0.502	-27.00978	13.23502
Idaho2010	-3.278132	9.781792	-0.34	0.738	-22.45746	15.9012
Idaho2011	6.268972	8.867739	0.71	0.480	-11.11816	23.6561
Idaho2012	5.673157	7.923792	0.72	0.474	-9.863161	21.20947
Idaho2013	-.298971	7.376224	-0.04	0.968	-14.76166	14.16372
Idaho2014	-4.957351	7.020338	-0.71	0.480	-18.72225	8.807551
Idaho2015	-.5080262	6.500638	-0.08	0.938	-13.25394	12.23789
Idaho2016	-3.406789	6.813995	-0.50	0.617	-16.76711	9.953532
Idaho2017	-2.312654	4.85723	-0.48	0.634	-11.83631	7.211003
Idaho2018	0	(omitted)				
Illinois2009	1.023479	8.842592	0.12	0.908	-16.31435	18.3613
Illinois2010	1.104646	8.329108	0.13	0.894	-15.22638	17.43567
Illinois2011	5.433236	7.40318	0.73	0.463	-9.082311	19.94878
Illinois2012	.2813645	6.370649	0.04	0.965	-12.20968	12.77241
Illinois2013	2.264581	5.787768	0.39	0.696	-9.083597	13.61276
Illinois2014	-5.326989	5.412402	-0.98	0.325	-15.93918	5.285204
Illinois2015	.3862167	5.24543	0.07	0.941	-9.89859	10.67102
Illinois2016	-4.003758	5.657161	-0.71	0.479	-15.09585	7.088337
Illinois2017	1.97882	3.690469	0.54	0.592	-5.257148	9.214789
Illinois2018	0	(omitted)				
Indiana2009	-.4604968	9.103303	-0.05	0.960	-18.3095	17.38851
Indiana2010	1.860027	8.813483	0.21	0.833	-15.42073	19.14078
Indiana2011	8.506104	7.803967	1.09	0.276	-6.795271	23.80748
Indiana2012	7.327311	6.662518	1.10	0.272	-5.736005	20.39063
Indiana2013	9.165019	5.942054	1.54	0.123	-2.485671	20.81571
Indiana2014	4.302048	5.587351	0.77	0.441	-6.65317	15.25727
Indiana2015	4.010971	5.365207	0.75	0.455	-6.508684	14.53063
Indiana2016	-1.18537	5.81497	-0.20	0.838	-12.58688	10.21614
Indiana2017	.9292654	3.785504	0.25	0.806	-6.493038	8.351569
Indiana2018	0	(omitted)				
Iowa2009	-2.497915	9.113122	-0.27	0.784	-20.36617	15.37034
Iowa2010	.4772894	8.512174	0.06	0.955	-16.21268	17.16726
Iowa2011	4.912702	7.60105	0.65	0.518	-9.990811	19.81621
Iowa2012	.8261764	6.482794	0.13	0.899	-11.88475	13.53711
Iowa2013	.9767643	6.040522	0.16	0.872	-10.86699	12.82052
Iowa2014	-8.147533	5.666581	-1.44	0.151	-19.2581	2.963031
Iowa2015	-5.981064	5.489709	-1.09	0.276	-16.74483	4.782706
Iowa2016	-9.513474	5.802509	-1.64	0.101	-20.89056	1.863608
Iowa2017	-1.655074	3.754834	-0.44	0.659	-9.017244	5.707095
Iowa2018	0	(omitted)				
Kansas2009	-3.385466	9.855834	-0.34	0.731	-22.70997	15.93904
Kansas2010	-5.317499	9.343489	-0.57	0.569	-23.63744	13.00244
Kansas2011	-.8694794	8.662193	-0.10	0.920	-17.85359	16.11464
Kansas2012	-3.587494	7.72268	-0.46	0.642	-18.72949	11.5545
Kansas2013	-3.15797	7.258706	-0.44	0.664	-17.39024	11.0743
Kansas2014	-9.156572	7.024201	-1.30	0.192	-22.92905	4.615904
Kansas2015	-2.393712	6.641993	-0.36	0.719	-15.41678	10.62936
Kansas2016	-5.215333	6.554049	-0.80	0.426	-18.06597	7.635305
Kansas2017	.6489666	4.482674	0.14	0.885	-8.140291	9.438224
Kansas2018	0	(omitted)				
Kentucky2009	3.949222	9.39059	0.42	0.674	-14.46307	22.36152
Kentucky2010	5.115637	8.857278	0.58	0.564	-12.25098	22.48226
Kentucky2011	9.918302	7.980196	1.24	0.214	-5.728608	25.56521
Kentucky2012	9.412676	6.965757	1.35	0.177	-4.245206	23.07056
Kentucky2013	10.65406	6.504477	1.64	0.102	-2.099383	23.4075
Kentucky2014	4.846278	6.023746	0.80	0.421	-6.964587	16.65714
Kentucky2015	3.169982	5.911602	0.54	0.592	-8.421	14.76096

Kentucky2016	-.2078793	5.968627	-0.03	0.972	-11.91067	11.49491
Kentucky2017	.1860465	4.001327	0.05	0.963	-7.659425	8.031518
Kentucky2018	0	(omitted)				
Louisiana2009	2.693094	10.73323	0.25	0.802	-18.35173	23.73792
Louisiana2010	7.564343	9.75112	0.78	0.438	-11.55485	26.68353
Louisiana2011	10.76051	8.441767	1.27	0.203	-5.791415	27.31243
Louisiana2012	1.523773	7.3277	0.21	0.835	-12.84378	15.89132
Louisiana2013	4.42142	6.731975	0.66	0.511	-8.778081	17.62092
Louisiana2014	2.825629	6.066933	0.47	0.641	-9.069913	14.72117
Louisiana2015	6.666662	5.74465	1.16	0.246	-4.596975	17.9303
Louisiana2016	-.0290903	6.056274	-0.00	0.996	-11.90373	11.84555
Louisiana2017	-.3816163	3.907894	-0.10	0.922	-8.043894	7.280661
Louisiana2018	0	(omitted)				
Maine2009	-5.537326	9.733877	-0.57	0.569	-24.62271	13.54806
Maine2010	-9.003148	9.029999	-1.00	0.319	-26.70843	8.70213
Maine2011	-3.927896	8.13395	-0.48	0.629	-19.87628	12.02048
Maine2012	-10.00108	7.106166	-1.41	0.159	-23.93426	3.932105
Maine2013	-5.185788	6.748025	-0.77	0.442	-18.41676	8.045183
Maine2014	-9.671357	6.541926	-1.48	0.139	-22.49823	3.155512
Maine2015	-1.038317	6.448973	-0.16	0.872	-13.68293	11.6063
Maine2016	-2.449759	6.557604	-0.37	0.709	-15.30737	10.40785
Maine2017	2.259003	4.020254	0.56	0.574	-5.623578	10.14159
Maine2018	0	(omitted)				
Maryland2009	5.635247	9.897904	0.57	0.569	-13.77175	25.04224
Maryland2010	-1.375699	9.451981	-0.15	0.884	-19.90836	17.15697
Maryland2011	2.133863	8.392644	0.25	0.799	-14.32174	18.58947
Maryland2012	-3.984457	7.328664	-0.54	0.587	-18.3539	10.38498
Maryland2013	-1.029784	6.510229	-0.16	0.874	-13.7945	11.73494
Maryland2014	-4.777422	6.330209	-0.75	0.450	-17.18917	7.634331
Maryland2015	-1.17909	6.147503	-0.19	0.848	-13.23261	10.87443
Maryland2016	-5.377323	5.925246	-0.91	0.364	-16.99506	6.24041
Maryland2017	-.5890337	3.840957	-0.15	0.878	-8.120065	6.941998
Maryland2018	0	(omitted)				
Massachusetts2009	11.15156	8.981101	1.24	0.214	-6.45784	28.76097
Massachusetts2010	8.650349	8.465917	1.02	0.307	-7.948923	25.24962
Massachusetts2011	10.63834	7.758531	1.37	0.170	-4.573952	25.85062
Massachusetts2012	2.854473	6.749625	0.42	0.672	-10.37964	16.08858
Massachusetts2013	3.398903	6.753287	0.50	0.615	-9.842386	16.64019
Massachusetts2014	-3.06643	6.557614	-0.47	0.640	-15.92406	9.791199
Massachusetts2015	3.277216	5.888094	0.56	0.578	-8.267674	14.82211
Massachusetts2016	-.5229841	5.653268	-0.09	0.926	-11.60745	10.56148
Massachusetts2017	.9325882	3.58842	0.26	0.795	-6.10329	7.968466
Massachusetts2018	0	(omitted)				
Michigan2009	-1.498383	8.918086	-0.17	0.867	-18.98423	15.98746
Michigan2010	-1.813042	8.487217	-0.21	0.831	-18.45408	14.82799
Michigan2011	1.297713	7.468305	0.17	0.862	-13.34553	15.94095
Michigan2012	-3.861573	6.284795	-0.61	0.539	-16.18428	8.461136
Michigan2013	.1819672	5.717744	0.03	0.975	-11.02891	11.39285
Michigan2014	-2.892834	5.4559	-0.53	0.596	-13.59031	7.804645
Michigan2015	2.71686	5.365775	0.51	0.613	-7.803909	13.23763
Michigan2016	-1.349649	5.705287	-0.24	0.813	-12.53611	9.836809
Michigan2017	1.593801	3.71541	0.43	0.668	-5.691069	8.87867
Michigan2018	0	(omitted)				
Minnesota2009	-10.97842	8.912528	-1.23	0.218	-28.45337	6.496528
Minnesota2010	-9.023267	8.504121	-1.06	0.289	-25.69745	7.650913
Minnesota2011	-2.224425	7.599284	-0.29	0.770	-17.12447	12.67563
Minnesota2012	-4.028003	6.408975	-0.63	0.530	-16.59419	8.538186
Minnesota2013	-1.906767	5.797651	-0.33	0.742	-13.27432	9.460791
Minnesota2014	-5.972045	5.618693	-1.06	0.288	-16.98872	5.044626
Minnesota2015	-3.523424	5.639282	-0.62	0.532	-14.58046	7.533616
Minnesota2016	-7.086553	5.789569	-1.22	0.221	-18.43826	4.265157
Minnesota2017	-1.96621	3.669194	-0.54	0.592	-9.160463	5.228044
Minnesota2018	0	(omitted)				
Mississippi2009	15.46612	10.01199	1.54	0.123	-4.164567	35.09682
Mississippi2010	13.21431	9.530636	1.39	0.166	-5.472578	31.90119
Mississippi2011	13.48408	8.555667	1.58	0.115	-3.291166	30.25933
Mississippi2012	5.739825	7.282985	0.79	0.431	-8.540052	20.0197
Mississippi2013	4.991243	6.693202	0.75	0.456	-8.132237	18.11472
Mississippi2014	-2.669603	6.392096	-0.42	0.676	-15.2027	9.863492
Mississippi2015	3.831993	6.177724	0.62	0.535	-8.280779	15.94477
Mississippi2016	-1.234355	6.065817	-0.20	0.839	-13.12771	10.659
Mississippi2017	3.673101	4.091893	0.90	0.369	-4.349946	11.69615
Mississippi2018	0	(omitted)				
Missouri2009	6.555014	9.243865	0.71	0.478	-11.5696	24.67962
Missouri2010	3.519478	8.747401	0.40	0.687	-13.63171	20.67066
Missouri2011	3.862353	7.802978	0.49	0.621	-11.43708	19.16179

Missouri2012	-.8033634	6.661735	-0.12	0.904	-13.86515	12.25842
Missouri2013	3.632314	6.028218	0.60	0.547	-8.187319	15.45195
Missouri2014	1.519256	5.598512	0.27	0.786	-9.457846	12.49636
Missouri2015	6.810911	5.531999	1.23	0.218	-4.035777	17.6576
Missouri2016	2.215616	5.763808	0.38	0.701	-9.085584	13.51682
Missouri2017	3.102803	3.760243	0.83	0.409	-4.269971	10.47558
Missouri2018	0	(omitted)				
Montana2009	-8.780518	9.914126	-0.89	0.376	-28.21932	10.65828
Montana2010	-6.613796	9.921887	-0.67	0.505	-26.06781	12.84022
Montana2011	-4.408592	9.307462	-0.47	0.636	-22.6579	13.84071
Montana2012	-2.977899	7.837534	-0.38	0.704	-18.34509	12.38929
Montana2013	3.654682	8.308556	0.44	0.660	-12.63605	19.94541
Montana2014	-.6359128	8.082488	-0.08	0.937	-16.48339	15.21156
Montana2015	1.263239	8.420268	0.15	0.881	-15.24653	17.77301
Montana2016	-4.659044	7.554013	-0.62	0.537	-19.47033	10.15224
Montana2017	-5.030335	4.801562	-1.05	0.295	-14.44484	4.384173
Montana2018	0	(omitted)				
Nebraska2009	-5.721438	10.00571	-0.57	0.567	-25.33981	13.89693
Nebraska2010	-8.219936	9.488969	-0.87	0.386	-26.82513	10.38525
Nebraska2011	-4.640977	8.977293	-0.52	0.605	-22.24291	12.96096
Nebraska2012	-7.909811	7.867183	-1.01	0.315	-23.33514	7.515515
Nebraska2013	-6.576083	7.13862	-0.92	0.357	-20.5729	7.420735
Nebraska2014	-12.84799	6.706882	-1.92	0.056	-25.9983	.3023091
Nebraska2015	-3.977291	6.778693	-0.59	0.557	-17.26839	9.313811
Nebraska2016	-4.716714	6.750276	-0.70	0.485	-17.9521	8.518671
Nebraska2017	4.114824	4.280229	0.96	0.336	-4.277496	12.50714
Nebraska2018	0	(omitted)				
Nevada2009	11.24802	14.0169	0.80	0.422	-16.23517	38.73121
Nevada2010	11.34787	12.98137	0.87	0.382	-14.10494	36.80067
Nevada2011	19.96976	14.13061	1.41	0.158	-7.736378	47.6759
Nevada2012	15.03797	10.07813	1.49	0.136	-4.722397	34.79833
Nevada2013	18.75167	11.20668	1.67	0.094	-3.221467	40.7248
Nevada2014	-.2412964	11.18474	-0.02	0.983	-22.17141	21.68881
Nevada2015	11.1099	11.46797	0.97	0.333	-11.37554	33.59535
Nevada2016	5.746187	9.696619	0.59	0.553	-13.26614	24.75852
Nevada2017	3.128	7.692743	0.41	0.684	-11.9553	18.2113
Nevada2018	0	(omitted)				
New Hampshire2009	-7.019052	9.251135	-0.76	0.448	-25.15791	11.11981
New Hampshire2010	-8.455439	8.508962	-0.99	0.320	-25.13911	8.228233
New Hampshire2011	-3.026604	7.93168	-0.38	0.703	-18.57839	12.52518
New Hampshire2012	-7.557946	7.787439	-0.97	0.332	-22.82691	7.711022
New Hampshire2013	-6.290812	7.41152	-0.85	0.396	-20.82271	8.241085
New Hampshire2014	-13.2569	6.386896	-2.08	0.038	-25.7798	-.7340036
New Hampshire2015	-7.770908	6.137559	-1.27	0.206	-19.80493	4.263111
New Hampshire2016	-8.305161	5.561681	-1.49	0.135	-19.21005	2.599724
New Hampshire2017	-1.475447	3.944791	-0.37	0.708	-9.210068	6.259174
New Hampshire2018	0	(omitted)				
New Jersey2009	-.9285133	9.138797	-0.10	0.919	-18.84711	16.99009
New Jersey2010	.8398032	8.550506	0.10	0.922	-15.92532	17.60493
New Jersey2011	6.717504	7.699736	0.87	0.383	-8.379503	21.81451
New Jersey2012	6.745684	6.445019	1.05	0.295	-5.891177	19.38255
New Jersey2013	7.646878	5.755029	1.33	0.184	-3.637108	18.93087
New Jersey2014	-.3086769	5.338104	-0.06	0.954	-10.77519	10.15784
New Jersey2015	.6379159	5.090353	0.13	0.900	-9.342829	10.61866
New Jersey2016	-4.625994	5.478368	-0.84	0.399	-15.36753	6.11554
New Jersey2017	-.2812778	3.566565	-0.08	0.937	-7.274306	6.71175
New Jersey2018	0	(omitted)				
New Mexico2009	-15.67847	11.40244	-1.38	0.169	-38.03544	6.678495
New Mexico2010	-14.34006	10.58424	-1.35	0.176	-35.09277	6.412644
New Mexico2011	-8.896735	8.947864	-0.99	0.320	-26.44097	8.647499
New Mexico2012	-7.759682	8.368142	-0.93	0.354	-24.16725	8.647882
New Mexico2013	-.3872095	10.61037	-0.04	0.971	-21.19115	20.41673
New Mexico2014	-5.947979	11.17322	-0.53	0.595	-27.85551	15.95955
New Mexico2015	-9.833054	10.11187	-0.97	0.331	-29.65958	9.993469
New Mexico2016	-14.11866	8.071843	-1.75	0.080	-29.94526	1.707945
New Mexico2017	-6.920112	5.551117	-1.25	0.213	-17.80429	3.964062
New Mexico2018	0	(omitted)				
New York2009	14.69268	8.888381	1.65	0.098	-2.734929	32.12028
New York2010	14.5434	8.252312	1.76	0.078	-1.637052	30.72385
New York2011	19.81107	7.276931	2.72	0.007	5.543064	34.07908
New York2012	15.17127	6.054452	2.51	0.012	3.300197	27.04234
New York2013	14.74473	5.46877	2.70	0.007	4.022013	25.46744
New York2014	6.20899	5.165309	1.20	0.229	-3.918723	16.3367
New York2015	7.97049	5.082644	1.57	0.117	-1.99514	17.93612
New York2016	.7694427	5.509248	0.14	0.889	-10.03264	11.57152
New York2017	2.601001	3.58511	0.73	0.468	-4.428388	9.630389

New York2018	0	(omitted)				
North Carolina2009	12.80043	9.081772	1.41	0.159	-5.00636	30.60722
North Carolina2010	11.22511	8.466171	1.33	0.185	-5.374665	27.82488
North Carolina2011	15.07524	7.56604	1.99	0.046	.2403677	29.9101
North Carolina2012	7.707019	6.378054	1.21	0.227	-4.798543	20.21258
North Carolina2013	6.091334	5.616236	1.08	0.278	-4.920518	17.10319
North Carolina2014	-2.119078	5.091668	-0.42	0.677	-12.1024	7.864246
North Carolina2015	1.868016	4.987868	0.37	0.708	-7.911784	11.64782
North Carolina2016	-2.970643	5.481164	-0.54	0.588	-13.71766	7.776372
North Carolina2017	.9837101	3.601798	0.27	0.785	-6.0784	8.04582
North Carolina2018	0	(omitted)				
North Dakota2009	-7.763835	10.18449	-0.76	0.446	-27.73274	12.20507
North Dakota2010	-6.698787	9.968509	-0.67	0.502	-26.24422	12.84664
North Dakota2011	-1.739637	10.34625	-0.17	0.866	-22.0257	18.54643
North Dakota2012	3.702683	8.901781	0.42	0.677	-13.7512	21.15656
North Dakota2013	9.85563	8.086811	1.22	0.223	-6.000323	25.71158
North Dakota2014	10.47034	7.930714	1.32	0.187	-5.079546	26.02023
North Dakota2015	3.993207	7.917766	0.50	0.614	-11.53129	19.51771
North Dakota2016	-2.452901	7.872153	-0.31	0.755	-17.88797	12.98217
North Dakota2017	-2.469072	4.981049	-0.50	0.620	-12.2355	7.297359
North Dakota2018	0	(omitted)				
Ohio2009	-9.726879	8.763123	-1.11	0.267	-26.90889	7.455131
Ohio2010	-8.0148	8.217978	-0.98	0.329	-24.12793	8.098335
Ohio2011	-1.409219	7.33106	-0.19	0.848	-15.78336	12.96492
Ohio2012	-1.775115	6.252835	-0.28	0.777	-14.03516	10.48493
Ohio2013	.2420724	5.698955	0.04	0.966	-10.93197	11.41611
Ohio2014	-6.2054	5.281058	-1.18	0.240	-16.56006	4.149263
Ohio2015	-2.359283	5.077234	-0.46	0.642	-12.31431	7.595739
Ohio2016	-6.349675	5.578914	-1.14	0.255	-17.28835	4.589
Ohio2017	-1.339602	3.635052	-0.37	0.713	-8.466912	5.787709
Ohio2018	0	(omitted)				
Oklahoma2009	10.20795	10.32005	0.99	0.323	-10.02675	30.44266
Oklahoma2010	5.401228	9.532174	0.57	0.571	-13.28867	24.09113
Oklahoma2011	3.424851	8.739575	0.39	0.695	-13.71099	20.56069
Oklahoma2012	-5.099371	7.444709	-0.68	0.493	-19.69634	9.4976
Oklahoma2013	-4.22231	7.083687	-0.60	0.551	-18.11142	9.6668
Oklahoma2014	-7.401103	6.488037	-1.14	0.254	-20.12231	5.320105
Oklahoma2015	2.242917	6.320281	0.35	0.723	-10.14937	14.6352
Oklahoma2016	.6520167	6.266191	0.10	0.917	-11.63421	12.93825
Oklahoma2017	5.465409	4.104672	1.33	0.183	-2.582694	13.51351
Oklahoma2018	0	(omitted)				
Oregon2009	-9.031016	9.268096	-0.97	0.330	-27.20314	9.141103
Oregon2010	-9.841953	8.713896	-1.13	0.259	-26.92744	7.243537
Oregon2011	-3.278269	7.811194	-0.42	0.675	-18.59382	12.03728
Oregon2012	-11.17076	6.844941	-1.63	0.103	-24.59176	2.250236
Oregon2013	-8.792438	6.119981	-1.44	0.151	-20.79199	3.207117
Oregon2014	-14.93881	5.843123	-2.56	0.011	-26.39553	-3.482096
Oregon2015	-7.613469	5.532649	-1.38	0.169	-18.46143	3.234493
Oregon2016	-8.166538	5.824278	-1.40	0.161	-19.5863	3.253226
Oregon2017	-2.31836	3.964122	-0.58	0.559	-10.09088	5.454164
Oregon2018	0	(omitted)				
Pennsylvania2009	5.523637	8.76098	0.63	0.528	-11.65417	22.70144
Pennsylvania2010	5.351401	8.15512	0.66	0.512	-10.63849	21.34129
Pennsylvania2011	10.98681	7.263164	1.51	0.130	-3.254207	25.22782
Pennsylvania2012	3.870142	6.189	0.63	0.532	-8.26474	16.00502
Pennsylvania2013	6.073	5.628507	1.08	0.281	-4.962913	17.10891
Pennsylvania2014	-3.706703	5.219379	-0.71	0.478	-13.94043	6.527025
Pennsylvania2015	1.081453	5.023882	0.22	0.830	-8.76896	10.93187
Pennsylvania2016	-4.393502	5.541181	-0.79	0.428	-15.25819	6.471188
Pennsylvania2017	.9313218	3.508385	0.27	0.791	-5.947631	7.810275
Pennsylvania2018	0	(omitted)				
Puerto Rico2009	24.72783	9.473785	2.61	0.009	6.152413	43.30325
Puerto Rico2010	22.24746	8.765308	2.54	0.011	5.061171	39.43376
Puerto Rico2011	32.54623	7.862313	4.14	0.000	17.13046	47.96201
Puerto Rico2012	23.35487	6.84221	3.41	0.001	9.939224	36.77051
Puerto Rico2013	18.05635	6.089809	2.97	0.003	6.115955	29.99675
Puerto Rico2014	4.260091	5.716368	0.75	0.456	-6.948093	15.46828
Puerto Rico2015	6.441902	5.398142	1.19	0.233	-4.142331	17.02613
Puerto Rico2016	1.176917	5.684959	0.21	0.836	-9.969682	12.32352
Puerto Rico2017	2.871668	3.680749	0.78	0.435	-4.345241	10.08858
Puerto Rico2018	0	(omitted)				
Rhode Island2009	19.45147	10.27129	1.89	0.058	-.6876293	39.59056
Rhode Island2010	10.01942	9.846508	1.02	0.309	-9.2868	29.32564
Rhode Island2011	10.56688	8.448836	1.25	0.211	-5.9989	27.13266
Rhode Island2012	6.026619	8.290584	0.73	0.467	-10.22887	22.28211
Rhode Island2013	7.65619	8.437892	0.91	0.364	-8.888133	24.20051

Rhode Island2014	.4987656	7.654616	0.07	0.948	-14.50978	15.50731
Rhode Island2015	4.462062	6.786439	0.66	0.511	-8.844228	17.76835
Rhode Island2016	-3.382086	5.658312	-0.60	0.550	-14.47644	7.712266
Rhode Island2017	1.174185	4.047101	0.29	0.772	-6.761038	9.109408
Rhode Island2018	0	(omitted)				
South Carolina2009	6.44428	9.49859	0.68	0.498	-12.17977	25.06833
South Carolina2010	6.719143	8.99439	0.75	0.455	-10.91632	24.3546
South Carolina2011	10.14888	7.938488	1.28	0.201	-5.41625	25.71402
South Carolina2012	5.099651	6.928647	0.74	0.462	-8.485469	18.68477
South Carolina2013	5.060615	6.327153	0.80	0.424	-7.345145	17.46638
South Carolina2014	2.257344	6.329391	0.36	0.721	-10.1528	14.66749
South Carolina2015	5.301692	6.233472	0.85	0.395	-6.920385	17.52377
South Carolina2016	-.1264129	6.345214	-0.02	0.984	-12.56759	12.31476
South Carolina2017	-.5722874	4.110504	-0.14	0.889	-8.631826	7.487251
South Carolina2018	0	(omitted)				
South Dakota2009	-9.732665	10.73696	-0.91	0.365	-30.78482	11.31949
South Dakota2010	-9.615376	10.50819	-0.92	0.360	-30.21897	10.98822
South Dakota2011	3.96606	9.399086	0.42	0.673	-14.46289	22.39501
South Dakota2012	1.433593	8.962161	0.16	0.873	-16.13867	19.00586
South Dakota2013	6.162056	7.899968	0.78	0.435	-9.32755	21.65166
South Dakota2014	-1.312567	7.926918	-0.17	0.868	-16.85501	14.22988
South Dakota2015	-1.349997	7.78336	-0.17	0.862	-16.61097	13.91097
South Dakota2016	-2.589828	7.504064	-0.35	0.730	-17.30318	12.12352
South Dakota2017	-3.6917	5.243459	-0.70	0.481	-13.97264	6.589242
South Dakota2018	0	(omitted)				
Tennessee2009	-2.80075	9.448861	-0.30	0.767	-21.3273	15.7258
Tennessee2010	-.985187	8.836786	-0.11	0.911	-18.31163	16.34126
Tennessee2011	3.400318	7.938803	0.43	0.668	-12.16543	18.96607
Tennessee2012	-1.885823	6.586508	-0.29	0.775	-14.8001	11.02846
Tennessee2013	-2.885655	6.058519	-0.48	0.634	-14.7647	8.99339
Tennessee2014	-8.121661	5.667903	-1.43	0.152	-19.23482	2.991496
Tennessee2015	-.7707139	5.593207	-0.14	0.890	-11.73741	10.19598
Tennessee2016	-1.159801	5.872578	-0.20	0.843	-12.67427	10.35467
Tennessee2017	1.587292	3.758789	0.42	0.673	-5.782631	8.957215
Tennessee2018	0	(omitted)				
Texas2009	-1.099104	8.839657	-0.12	0.901	-18.43117	16.23297
Texas2010	-4.922518	8.312809	-0.59	0.554	-21.22159	11.37655
Texas2011	-.3681651	7.433797	-0.05	0.961	-14.94374	14.20741
Texas2012	-2.842526	6.239097	-0.46	0.649	-15.07563	9.390582
Texas2013	-.1279146	5.655204	-0.02	0.982	-11.21617	10.96034
Texas2014	-3.42676	5.313459	-0.64	0.519	-13.84495	6.991432
Texas2015	.4208738	5.254564	0.08	0.936	-9.881842	10.72359
Texas2016	-3.948587	5.628589	-0.70	0.483	-14.98466	7.087486
Texas2017	1.842724	3.701356	0.50	0.619	-5.414589	9.100038
Texas2018	0	(omitted)				
Utah2009	-1.885964	12.45084	-0.15	0.880	-26.29854	22.52661
Utah2010	-9.538943	11.42759	-0.83	0.404	-31.94522	12.86733
Utah2011	-4.461769	9.234278	-0.48	0.629	-22.56758	13.64404
Utah2012	-3.240259	7.726887	-0.42	0.675	-18.3905	11.90998
Utah2013	.1658864	7.920129	0.02	0.983	-15.36325	15.69502
Utah2014	-5.887811	8.716053	-0.68	0.499	-22.97753	11.20191
Utah2015	.7648804	9.31709	0.08	0.935	-17.5033	19.03306
Utah2016	-4.022507	9.886655	-0.41	0.684	-23.40745	15.36243
Utah2017	.6750834	6.60741	0.10	0.919	-12.28018	13.63035
Utah2018	0	(omitted)				
Vermont2009	-11.2764	12.76149	-0.88	0.377	-36.29809	13.74528
Vermont2010	-14.3395	10.02844	-1.43	0.153	-34.00245	5.32344
Vermont2011	-7.751937	9.194642	-0.84	0.399	-25.78003	10.27616
Vermont2012	-7.324913	6.805421	-1.08	0.282	-20.66842	6.018597
Vermont2013	-3.81994	7.802203	-0.49	0.624	-19.11786	11.47798
Vermont2014	-7.612274	6.721299	-1.13	0.257	-20.79084	5.566297
Vermont2015	-1.387396	7.666546	-0.18	0.856	-16.41933	13.64454
Vermont2016	-1.515054	6.345051	-0.24	0.811	-13.95591	10.9258
Vermont2017	1.420131	3.834244	0.37	0.711	-6.097738	8.938001
Vermont2018	0	(omitted)				
Virginia2009	3.324263	9.250704	0.36	0.719	-14.81375	21.46228
Virginia2010	1.2715	8.708471	0.15	0.884	-15.80335	18.34635
Virginia2011	7.287354	7.997533	0.91	0.362	-8.39355	22.96826
Virginia2012	3.719319	6.895098	0.54	0.590	-9.800021	17.23866
Virginia2013	3.903523	6.492316	0.60	0.548	-8.826075	16.63312
Virginia2014	-4.739113	5.883034	-0.81	0.421	-16.27408	6.795856
Virginia2015	-3.069062	5.472723	-0.56	0.575	-13.79953	7.661402
Virginia2016	-6.506851	5.665653	-1.15	0.251	-17.6156	4.601894
Virginia2017	-.4168372	3.820449	-0.11	0.913	-7.90766	7.073985
Virginia2018	0	(omitted)				
Washington2009	6.415919	9.050509	0.71	0.478	-11.32957	24.16141

Washington2010	3.990801	8.550776	0.47	0.641	-12.77486	20.75646
Washington2011	8.88254	7.390527	1.20	0.229	-5.608197	23.37328
Washington2012	4.603741	6.365127	0.72	0.470	-7.876475	17.08396
Washington2013	5.24738	5.88858	0.89	0.373	-6.298463	16.79322
Washington2014	-2.673475	5.626803	-0.48	0.635	-13.70605	8.359097
Washington2015	1.491205	5.49762	0.27	0.786	-9.288077	12.27049
Washington2016	-3.875594	5.777247	-0.67	0.502	-15.20314	7.451955
Washington2017	.8001055	3.852588	0.21	0.835	-6.753731	8.353942
Washington2018	0	(omitted)				
West Virginia2009	17.57472	9.259992	1.90	0.058	-.5815095	35.73095
West Virginia2010	16.30022	8.986097	1.81	0.070	-1.318975	33.91942
West Virginia2011	20.13178	8.575042	2.35	0.019	3.318542	36.94501
West Virginia2012	17.00337	7.541272	2.25	0.024	2.21706	31.78967
West Virginia2013	13.10875	6.786474	1.93	0.053	-.197605	26.41511
West Virginia2014	3.549511	6.22208	0.57	0.568	-8.650231	15.74925
West Virginia2015	2.144579	6.018955	0.36	0.722	-9.656892	13.94605
West Virginia2016	-1.836323	6.109251	-0.30	0.764	-13.81484	10.14219
West Virginia2017	.789758	4.003406	0.20	0.844	-7.059791	8.639307
West Virginia2018	0	(omitted)				
Wisconsin2009	-5.512709	8.853494	-0.62	0.534	-22.87191	11.84649
Wisconsin2010	-2.153033	8.37845	-0.26	0.797	-18.58081	14.27474
Wisconsin2011	6.782095	7.483894	0.91	0.365	-7.891707	21.4559
Wisconsin2012	4.098282	6.274984	0.65	0.514	-8.205189	16.40175
Wisconsin2013	5.785796	5.837844	0.99	0.322	-5.660568	17.23216
Wisconsin2014	-.668148	5.373383	-0.12	0.901	-11.20383	9.867539
Wisconsin2015	4.299664	5.362412	0.80	0.423	-6.214511	14.81384
Wisconsin2016	-2.826194	5.707746	-0.50	0.621	-14.01747	8.365084
Wisconsin2017	1.121963	3.702761	0.30	0.762	-6.138105	8.38203
Wisconsin2018	0	(omitted)				
Wyoming2009	0	(omitted)				
Wyoming2010	0	(omitted)				
Wyoming2011	0	(omitted)				
Wyoming2012	0	(omitted)				
Wyoming2013	0	(omitted)				
Wyoming2014	0	(omitted)				
Wyoming2015	0	(omitted)				
Wyoming2016	0	(omitted)				
Wyoming2017	0	(omitted)				
Wyoming2018	0	(omitted)				
_cons	313.2453	32.97281	9.50	0.000	248.5949	377.8956
sigma_u	52.761728					
sigma_e	21.882087					
rho	.85323914	(fraction of variance due to u_i)				

TOTAL NUMBER OF MISSING OBSERVATIONS BY DEPENDENT VARIABLE

National reporting on death rates from NCHS data is conventionally based on reported data, with the counties (and states) with missing data, excluded from calculation of area-level averages. The US comprises a total of 56 states & territories, including 51 states (District of Columbia included) and 5 territories (American Samoa, Guam, Northern Mariana Islands, Puerto Rico, and US Virgin Islands). Data on CVD mortality rate amounted to a total of 31,017 observations and covered a total of 3,152 counties and 53 states & territories over the ten-year period. A total of 3 territories had no (zero) counties reporting data on CVD deaths over the entire ten-year period, including American Samoa, Northern Mariana Islands, and US Virgin Islands. Data on opioid use mortality rate amounted to a total of 3,273 observations and covered a total of 554 counties and 48 states & territories over the ten-year period. A total of 8 states & territories had no (zero) counties reporting data on opioid use deaths over the entire ten-year period, including 3 states (Montana, South Dakota, Wyoming) and 5 territories (American Samoa, Guam, Northern Mariana Islands, Puerto Rico, US Virgin Islands).

Although the difference between total county-level observations for CVD mortality and opioid use mortality (i.e., 31,017 vs. 3,273 observations respectively), may raise questions about the completeness of the opioid use mortality data, it would be relevant to note that the SDoH database draws upon the official national source of data on opioid mortality, i.e., the CDC, which in turn oversees the NCHS, i.e., the national center responsible for maintaining all individual death certificate data submitted by states. Importantly, our descriptive statistics on CVD and opioid use death rates (summarized in Figures 1-4), serve to confirm that our calculations related to national, regional, and state-level trends and averages for both CVD and opioid use death rates, are fully consistent with publicly reported statistics on CVD and opioid use death rates across all three area-levels of interest in the US (national, regional, and state), over the same time-period.

METHOD FOR CALCULATING IMPACT OF SIGNIFICANT PREDICTORS ON SAMPLE MEAN MORTALITY RATES (TABLES 3 AND TABLE 5)

Turning to the primary findings for CVD death rate, based on the regression coefficient value for ACS_PCT_ARMED_FORCES indicated in Table 2, Column 2 for county and year fixed-effects, a 1% increase in 'percentage of civilian population in armed forces,' significantly predicts an increase in CVD death rate by 1.63 per 100,000 ($p<0.01$). This also represents a 0.64% increase in the mean CVD death rate of 250.73 per 100,000 (in the sample), i.e., (1.63/250.73)*100. Another way of estimating impact of a change in ACS_PCT_ARMED_FORCES on the mean CVD death rate, would be as follows:

- If a 1% increase in ACS_PCT_ARMED_FORCES is significant in increasing CVD death rate by 1.63 per 100,000 and correspondingly, increasing the mean CVD death rate (in the sample), by **0.64%**, then what would the impact of a one standard deviation (SD) change in ACS_PCT_ARMED_FORCES be, on the mean CVD death rate?
- Given SD of ACS_PCT_ARMED_FORCES is 1.66, this question translates to: 'what would the impact of a 1.66% increase in 'percentage of civilian population in armed forces' be, on the mean CVD death rate?' The answer to this would be [(1.63*1.66)/250.73]*100, which translates to a **1.07%** increase in the mean CVD death rate.
- For practical purposes therefore, 1.63*1.66 (i.e., coefficient value*SD), represents the effect size. In other words, the impact of significant SDoH predictors on mean CVD (or opioid use) death rates could be calculated based on both coefficient value and effect size.

This practical approach to calculating the impact of significant predictor variables on the dependent variable (sample mean) based on both coefficient value and effect size, has been leveraged in a vast number of health economic research studies that have utilized similar county-level panel datasets and fixed-effects regression techniques. For the purposes of this study, the aforementioned approach was used to calculate percent change in mean CVD death rate and mean opioid use death rate (in the sample), for all significant SDoH predictors in both specifications. Results are summarized in Table 3 and Table 5.
